# A Small Molecule Inhibitor of Erg251 Makes Fluconazole Fungicidal by Inhibiting the Synthesis of the 14α-Methylsterols

**DOI:** 10.1128/mbio.02639-22

**Published:** 2022-12-08

**Authors:** Hui Lu, Wanqian Li, Malcolm Whiteway, Hongkang Wang, Shuo Zhu, Zhe Ji, Yanru Feng, Lan Yan, Ting Fang, Liping Li, Tingjunhong Ni, Xiaolong Zhang, Quanzhen Lv, Zichao Ding, Lijuan Qiu, Dazhi Zhang, Yuanying Jiang

**Affiliations:** a Department of Pharmacy, Shanghai Tenth People's Hospital, School of Medicine, Tongji University, Shanghai, China; b School of Pharmacy, Naval Medical University, Shanghai, China; c Department of Biology, Concordia University, Montreal, Quebec, Canada; d Department of Physiology and Pharmacology, School of Basic Medicine and Clinical Pharmacy, China Pharmaceutical University, Nanjing, China; Mass General Hospital

**Keywords:** fluconazole tolerance, Erg251, small molecule inhibitor, fluconazole adjuvant, *Candida albicans*

## Abstract

Fluconazole (FLC) is widely used to prevent and treat invasive fungal infections. However, FLC is a fungistatic agent, allowing clinical FLC-susceptible isolates to tolerate FLC. Making FLC fungicidal in combination with adjuvants is a promising strategy to avoid FLC resistance and eliminate the persistence and recurrence of fungal infections. Here, we identify a new small molecule compound, CZ66, that can make FLC fungicidal. The mechanism of action of CZ66 is targeting the C-4 sterol methyl oxidase, encoded by the *ERG251* gene, resulting in decreased content of sterols with the 14α-methyl group and ultimately eliminating FLC tolerance of Candida albicans. CZ66 most likely interacts with Erg251 through residues Glu195, Gly206, and Arg241. Establishing Erg251 as a synergistic lethal target protein of FLC should direct research to identify specific small molecule inhibitors of 14α-methylsterol synthesis and open the way to abolishing fungal FLC tolerance.

## INTRODUCTION

Candida albicans is the most common pathogenic fungus that can cause fatal invasive infections in immunodeficient individuals, such as AIDS patients, cancer patients, and organ transplant recipients (1). As a representative antifungal azole, fluconazole (FLC) is clinically the most widely used antifungal drug because of its broad spectrum, impressive safety profile, and availability in oral and intravenous formulations (2). However, FLC is a fungistatic drug that inhibits growth but does not kill the pathogenic fungus, leading to FLC tolerance of C. albicans, which is characterized by FLC-susceptible C. albicans strains that can grow slowly in the presence of FLC at concentrations above the MIC (3). While the rate of FLC resistance in C. albicans is generally low (less than 1%) (4), FLC often fails to treat invasive fungal infections caused by susceptible C. albicans isolates. Although the discordance between overall treatment outcome and low levels of clinical resistance is likely to be multifactorial (5), FLC tolerance of C. albicans is important because persistent candidemia is associated with high FLC tolerance levels of C. albicans isolates (6). In addition, FLC tolerance has been proposed to increase acquired FLC resistance in C. albicans (7). Therefore, making FLC fungicidal and eliminating FLC tolerance in C. albicans is an important goal for successfully treating invasive fungal infections.

It is reported that FLC tolerance is due to phenotypic heterogeneity rather than a genetic alteration in the subpopulation and is stable for a given C. albicans strain (6). Functional calcineurin (8), heat shock protein 90 (Hsp90) (9), the target of rapamycin (TOR) signaling pathway (6), the unfolded protein response (UPR) pathway (6), protein kinase C (PKC) signaling (9), ADP ribosylation factor (10), and iron homeostasis (6) are crucial for the survival of C. albicans exposed to FLC. However, how these factors increase the fungicidal activity of FLC remains to be determined. The inhibition of C14α-demethylase (Erg11) by FLC reduces the intracellular levels of ergosterol and increases 14α-methylsterol levels (11). 14α-Methylsterols allow C. albicans to survive in the presence of FLC, but this change in sterol composition results in growth arrest (12–14) and increased sensitivity to environmental stresses. Therefore, we hypothesized that 14α-methylsterols contribute to FLC tolerance.

**FIG 1:**
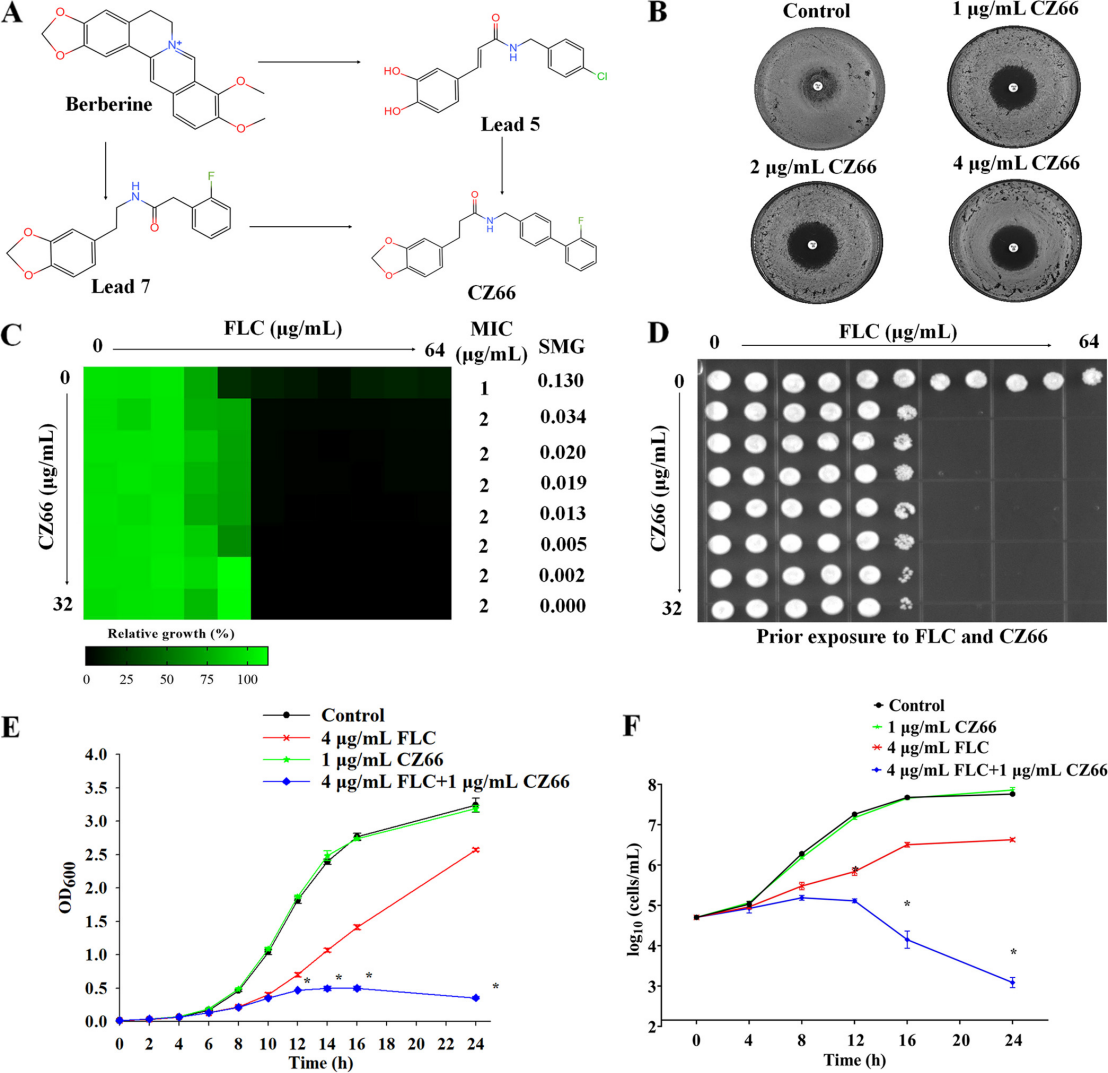
CZ66 makes FLC fungicidal. (A) Chemical structural formula and design of CZ66. (B) Disk diffusion assays showed that different concentrations of CZ66 (1, 2, and 4 μg/mL) made the zones of inhibition of 25 μg FLC clear. In brief, cells (2 × 10^5^ cells) were spread onto YPD plates or YPD-containing CZ66 plates. A single 25-μg FLC disk (6 mm) (Liofilchem, Italy) was placed in the center of each plate. Plates were then incubated at 30°C for 48 h before plates were photographed. (C) Dose-matrix titration assays showed that CZ66 (concentration range from 0.5 μg/mL to 32 μg/mL) abolished FLC tolerance of C. albicans incubated in YPD medium at 30°C for 48 h. (D) Cells from the dose-matrix titration assays were spotted onto YPD medium and incubated at 30°C for 48 h before plates were photographed. (E) Growth inhibition curve assays. Overnight C. albicans cultures were inoculated into 100 mL of fresh YPD medium with 4 μg/mL FLC, 1 μg/mL CZ66, or 4 μg/mL FLC and 1 μg/mL CZ66 or without any compound (control) to a final OD_600_ of 0.015 to 0.020. The optical density was measured every 2 h until the stationary phase of the growth curve was reached. *, *P* < 0.05 by *t* test for the group treated with 4 μg/mL FLC plus 1 μg/mL CZ66 compared to the group treated with 4 μg/mL FLC. (F) Time-kill curve assays. Overnight C. albicans cultures were inoculated into 100 mL of fresh YPD medium with 4 μg/mL FLC, 1 μg/mL CZ66, or 4 μg/mL FLC and 1 μg/mL CZ66 or without any compound (control) to a final optical density of around 1 × 10^5^ cells/mL. Every 2 h of exposure, triplicate-sample aliquots were removed, serially diluted, and plated on an appropriate drug-free agar medium. *, *P* < 0.05 by *t* test for the group treated with 4 μg/mL FLC plus 1 μg/mL CZ66 compared to the group treated with 4 μg/mL FLC.

Synergistic combinations of FLC and an adjuvant drug are promising therapies, as the combinations can be fungicidal against C. albicans (2). These adjuvants work by inhibiting proteins that play an essential role in FLC tolerance; inhibitors of calcineurin (cyclosporine and FK506), Hsp90 (geldanamycin), TOR (rapamycin), and a guanine nucleotide exchange factor (brefeldin A) can make FLC fungicidal (6, 10, 15, 16). However, the clinical applications of these drug combinations are limited because of their high levels of toxicity (17). Therefore, adjuvants are needed that inactivate new synergistic targets to make FLC fungicidal. Our previous studies found that berberine and its derivates (lead compounds 5 and 7) can significantly enhance the antifungal activity of FLC against FLC-resistant C. albicans (18, 19). Based on the structure of these compounds, we designed and synthesized a series of 3-(benzo [d] [1,3] dioxol-5-yl)-*N*-(substituted benzyl) propanamides and then evaluated their synergistic antifungal activity with FLC against FLC-resistant C. albicans. We found that one of these compounds, named CZ66 (ZINC database no. 1772579137) (Fig. 1A), had the strongest antifungal activity against FLC-resistant C. albicans in combination with FLC, as the fractional inhibitory concentration index of FLC and CZ66 is 0.063 (20). However, the target of CZ66 was unclear.

Here, we explored FLC and CZ66 synergistic fungicidal effects. Interestingly, we found that CZ66 specifically enhanced the antifungal activity of azoles and that an *ERG11* gene null mutant (*erg11*Δ/*erg11*Δ) is hypersensitive to CZ66. We further found that the synergistic fungicidal effect of CZ66 and FLC depends on the disruptive impact of CZ66 on ergosterol biosynthesis and confirmed that Erg251 is a potential target of CZ66. Erg251, a C-4 sterol methyl oxidase, catalyzes the first three steps required to remove two C-4 methyl groups from an intermediate in ergosterol biosynthesis (21). CZ66 most likely interacts with Erg251 through Glu195, Gly206, and Arg241. In the presence of FLC, the *ERG251* gene null mutant (*erg251*Δ/*erg251*Δ) displayed no FLC tolerance due to the inhibition of the synthesis of 14α-methylsterols. This study reveals that Erg251 is a synergistic lethal target of FLC and might be suitable for developing small molecule drugs that could make FLC fungicidal in practice.

## RESULTS

### CZ66 makes FLC fungicidal.

C. albicans shows tolerance to FLC, allowing growth above the MIC value of FLC (22). Due to the tolerance of C. albicans to FLC, disk diffusion assays showed noticeable growth of cells in the zone of inhibition of 25 μg FLC on YPD (yeast extract-peptone-dextrose) plates incubated at 30°C for 48 h (6, 23). In contrast, the zones of inhibition for 25-μg FLC treatments were clear on YPD plates containing 1, 2, or 4 μg/mL CZ66, respectively (Fig. 1B). Dose-matrix titration assays showed that CZ66, in concentration ranges from 0.5 μg/mL to 32 μg/mL, has no antifungal activity by itself but can abolish FLC tolerance of C. albicans: that is, CZ66 significantly reduced super-MIC growth (SMG) of C. albicans in the presence of FLC (Fig. 1C). We spotted C. albicans cells from the dose-matrix titration assays onto YPD solid medium. We found that cells treated with CZ66 plus FLC could not recover on YPD solid medium, while cells treated with FLC could (Fig. 1D), indicating that CZ66 makes FLC fungicidal. This synthetic lethality of CZ66 and FLC depended on the FLC dose (≥4 μg/mL) but not the CZ66 dose because different concentrations of CZ66 lead to the same fungicidal activity of FLC (Fig. 1C and D). Growth inhibition assays showed that 1 μg/mL CZ66 alone does not affect the growth of C. albicans. CZ66 (1 μg/mL) did not enhance the antifungal activity of FLC (4 μg/mL) when FLC did not significantly inhibit the growth of C. albicans during the lag phase (earlier than 6 h). However, when C. albicans enters early logarithmic growth in the presence of 4 μg/mL FLC (later than 12 h), the combination of FLC and CZ66 shows a stronger inhibitory effect than FLC alone (Fig. 1E). Similarly, after C. albicans cells were treated for more than 12 h, the combination of FLC and CZ66 showed a fungicidal effect as determined in time-kill curve assays (Fig. 1F). All of these results demonstrated that CZ66 could eliminate the tolerance of C. albicans to FLC and turn FLC from fungistatic to fungicidal.

### CZ66 specifically enhanced the antifungal effect of azoles.

**FIG 2:**
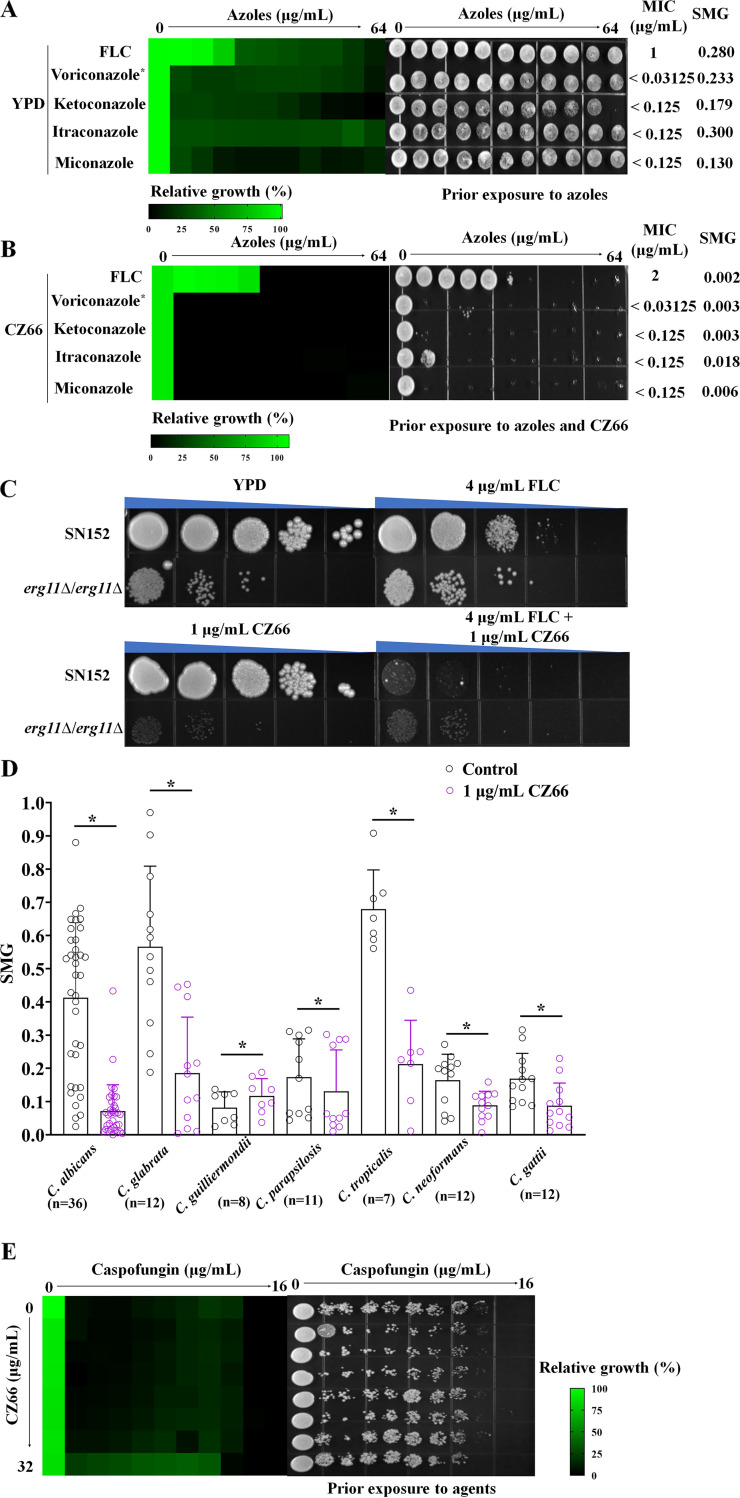
The synergistic lethality of FLC and CZ66 depends on the inhibition of the Erg11 activity. (A) The sensitivities of C. albicans to FLC, voriconazole, ketoconazole, itraconazole, and miconazole were tested by the broth microdilution assays in YPD medium incubated at 30°C for 48 h (left). Cells from the broth microdilution assays were spotted onto YPD medium and incubated at 30°C for 48 h before the plate was photographed (right). *, the initial concentration of voriconazole was 16 μg/mL. (B) The sensitivities of C. albicans to FLC, voriconazole, ketoconazole, itraconazole, and miconazole were tested by broth microdilution assays in YPD medium with 1 μg/mL CZ66 incubated at 30°C for 48 h (left). Cells from the broth microdilution assays were spotted onto YPD medium and incubated at 30°C for 48 h before the plate was photographed (right). *, the initial concentration of voriconazole was 16 μg/mL. (C) CZ66 has an inhibitory effect on the *erg11*Δ/*erg11*Δ mutant. An overnight C. albicans culture was adjusted to 1 × 10^7^ cells/mL. It then was spotted (1:10 dilution) onto a solid YPD medium with 4 μg/mL FLC, 1 μg/mL CZ66, or 4 μg/mL FLC, and 1 μg/mL CZ66 or without any compound and photographed after 48 h of incubation at 30°C. (D) CZ66 can reduce the tolerance level of FLC of *Candida* and Cryptococcus species. *, *P* < 0.05 by *t* test for the group treated with 1 μg/mL CZ66 compared to the control group. (E) Dose-matrix titration assays showed that CZ66 (concentration range from 0.5 μg/mL to 32 μg/mL) could not abolish caspofungin tolerance of C. albicans incubated in YPD medium at 30°C for 48 h. Cells from the dose-matrix titration assays were spotted onto YPD medium and incubated at 30°C for 48 h before the plate was photographed.

We further investigated the synergistic lethal effect of CZ66 with different azoles, including voriconazole, ketoconazole, itraconazole, and miconazole. We found that CZ66 made all of these azoles fungicidal (Fig. 2A and B). Azoles block the ergosterol synthetic pathway by directly inhibiting a C14α-demethylase encoded in C. albicans by the *ERG11* gene (C5_00660C_A), which catalyzes the oxidative removal of the 14α-methyl group from eburicol (11, 24). Since CZ66 and azoles have a synergistic fungicidal activity, we hypothesized that the *ERG11* null mutant (*erg11*Δ/*erg11*Δ) strain would be hypersensitive to CZ66. Therefore, we constructed an *erg11*Δ/*erg11*Δ strain and found that this strain showed strong sensitivity to CZ66 (Fig. 2C), which is consistent with CZ66 making azoles fungicidal and supported the synergistic lethal effect of CZ66 and azoles based on inhibition of the activity of Erg11.

To examine whether CZ66’s inhibition of FLC tolerance is conserved across other C. albicans strains and pathogenic *Candida* species, we tested combinations of FLC plus 1 μg/mL CZ66 in clinical isolates of C. albicans (*n* = 36), C. glabrata (*n* = 12), C. guilliermondii (*n* = 8), C. parapsilosis (*n* = 11), and C. tropicalis (*n* = 7). The clinical isolates of C. albicans have a high tolerance to FLC (Fig. 2D), and C. glabrata, C. parapsilosis, and *C. tropicalis* showed tolerance in the presence of FLC (Fig. 2D) (10). A 1-μg/mL concentration of CZ66 significantly reduced FLC tolerance in these fungi (Fig. 2D). However, CZ66 did not synergize with FLC in C. guilliermondii (Fig. 2D). To further investigate CZ66 as an adjuvant abrogating FLC tolerance in non-*Candida* human fungal pathogens, we asked whether CZ66 would synergize with FLC against Cryptococcus species. We found that CZ66 significantly decreased SMG values of clinical isolates of Cryptococcus neoformans (*n* = 12) and Cryptococcus gattii (*n* = 12) (Fig. 2D).

Interestingly, CZ66 did not enhance the antifungal activities of other ergosterol synthesis inhibitors, such as cerulenin (an inhibitor of 3-hydroxy-3-methylglutaryl coenzyme A) (25) (see Fig. S1A in the supplemental material), fluvastatin (an inhibitor of Hmg1) (26) (Fig. S1B), or terbinafine (an inhibitor of Erg1) (27) (Fig. S1C). Damage to the cell wall can increase the sensitivity of C. albicans to FLC (2). However, broth microdilution assays showed that CZ66 could not eliminate the caspofungin tolerance of C. albicans (Fig. 2E). Similarly, CZ66 did not enhance the antifungal activity of Congo red (Fig. S1D), which causes cell wall integrity stress (28). These results suggested that CZ66 does not aggravate C. albicans cell wall damage.

10.1128/mbio.02639-22.2 FIG S1 Dose-matrix titration assays (incubated in a YPD medium at 30°C for 24 h) were used to evaluate the synergistic effect of CZ66 and antifungal agents, including (A) cerulenin, (B) fluvastatin, (C) terbinafine, (D) Congo red, (E) amphotericin B, (F) SDS, (G) rapamycin, (H) myriocin, and (I) 5-fluorocytosine. (J) Evaluation of the sensitivity of C. albicans to CZ66 in YPDextrose, YPFructose, YPGalactose, YPGlcNAc, YPMannose, YPSucrose, YPGlycerol, and YPEthanol media using the broth microdilution assays incubated at 30°C for 24 h. Download FIG S1, TIF file, 0.3 MB. Copyright © 2022 Lu et al. 2022 Lu et al. https://creativecommons.org/licenses/by/4.0/ This content is distributed under the terms of the Creative Commons Attribution 4.0 International license.

The destruction of cell membrane integrity can also enhance the antifungal activity of FLC (15). We found that CZ66 was unable to synergize with the antifungal activities of amphotericin B (Fig. S1E) and sodium dodecyl sulfate (SDS) (Fig. S1F), which can disrupt the cell membrane integrity (29, 30). In addition, sphingolipids also play an important role in C. albicans resistance to FLC (31). We found that CZ66 did not enhance the antifungal activities of rapamycin (Fig. S1G), which is an inhibitor of the TOR pathway and able to inhibit sphingolipid synthesis (32), or of myriocin (Fig. S1H), which is an inhibitor of serine-palmitoyl-transferase that plays a vital role in sphingolipid synthesis (33). Finally, CZ66 did not enhance the antifungal effect of 5-fluorocytosine (Fig. S1I), which causes RNA miscoding and inhibits DNA synthesis (34).

### The synergistic fungicidal effect of CZ66 and FLC depends on the disruptive impact of CZ66 on ergosterol biosynthesis.

**FIG 3:**
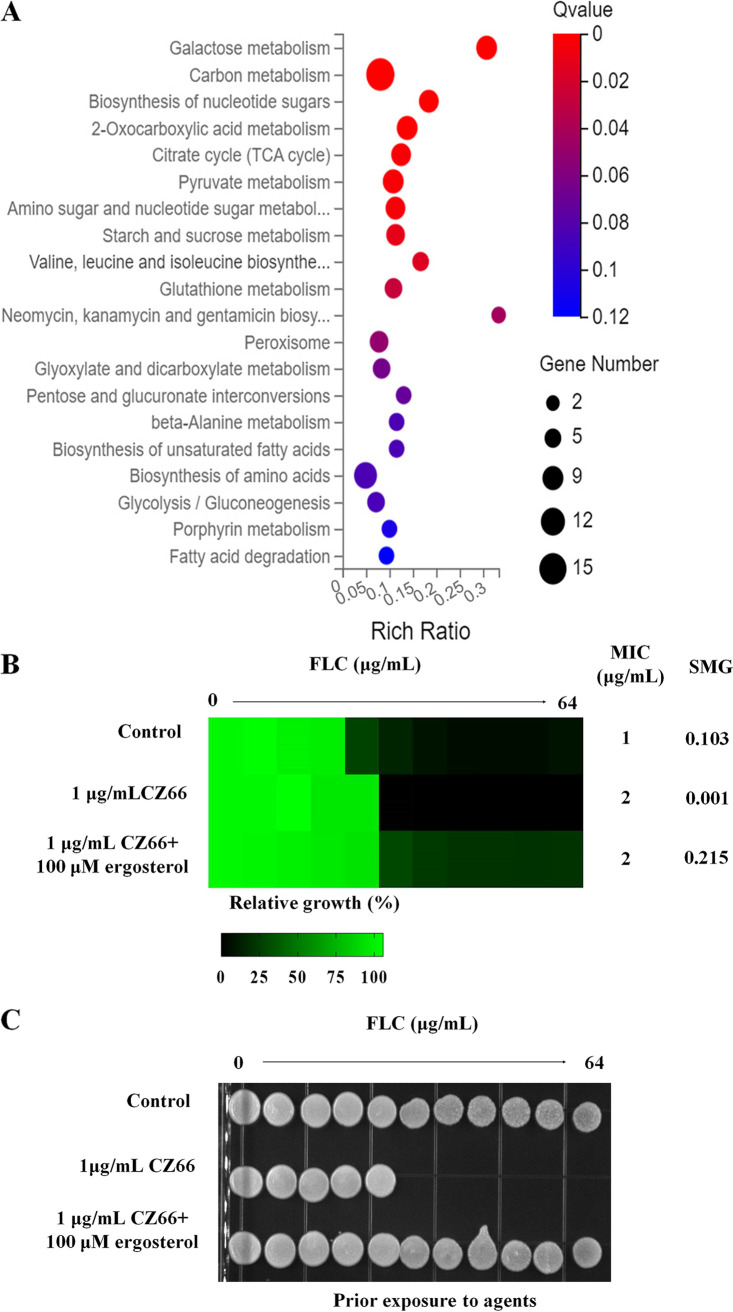
CZ66 has an inhibitory effect on ergosterol synthesis. (A) CZ66 may mainly affect the biological processes of carbon, amino acid, and fatty acid metabolism using KEGG analysis through RNA-seq analysis of *C. albicans* cells treated with 4 μg/mL FLC and cells treated with 4 μg/mL FLC plus 1 μg/mL CZ66. (B) The broth microdilution assays indicated that 100 μM ergosterol counteracted the synergistic lethal effect of CZ66 (1 μg/mL) and FLC (4 μg/mL) in YPD medium incubated at 30°C for 48 h. (C) Cells from the broth microdilution assays were spotted onto YPD medium and incubated at 30°C for 48 h before the plate was photographed.

To investigate the mechanism of the synergistic lethality of CZ66 and FLC, we carried out a transcriptome sequencing (RNA-seq) experiment. C. albicans cells were divided into two groups and treated with 4 μg/mL FLC and 4 μg/mL FLC plus 1 μg/mL CZ66 for 12 h, respectively. KEGG analysis showed that the expression of genes affected by CZ66 was enriched in sugar metabolic signaling pathways (Fig. 3A). We speculated that CZ66 enhances the antifungal effect of FLC by interfering with the carbon metabolism of C. albicans and therefore tested the inhibitory effect of CZ66 on the growth of C. albicans in different carbon sources, including fermentable carbon sources (dextrose, fructose, galactose, *N*-acetyl-d-glucosamine [GlcNAc], mannose, and sucrose) and nonfermentable carbon sources (glycerol and ethanol). However, we found that CZ66 did not affect the growth of C. albicans on the different carbon sources (Fig. S2J), which did not support our conjecture that the synergistic fungicidal effect of CZ66 and FLC depends on the inhibitory effect of CZ66 on carbon source metabolism. KEGG analysis also showed that CZ66 might affect amino acid metabolism in C. albicans (Fig. 3A). The TOR signaling pathway controls cell growth in response to nutrients, particularly amino acids (35). However, CZ66 did not affect the growth rate of C. albicans (Fig. 1E) or enhance the antifungal activities of rapamycin (Fig. S1G). These results indicated that CZ66-affected expression of genes related to amino acid metabolism might be the by-product of CZ66 affecting other biosynthetic pathways.

10.1128/mbio.02639-22.3 FIG S2 (A) Rhodamine 6G efflux experiment without glucose (left) and with glucose (right). (B) We used the broth microdilution assays (incubated in YPD medium at 30°C for 48 h) to evaluate the MIC value of FLC of the SN152 strain in YPD medium with 1 μg/mL CZ66, 1 μg/mL cyclosporine, 1 μg/mL cyclosporine and 1 μg/mL CZ66, or without any compound (as control) (left). Cells from the broth microdilution assays were spotted onto YPD medium and incubated at 30°C for 48 h before the plate was photographed (right). (C) We used the broth microdilution assays (incubated in YPD medium at 30°C for 48 h) to evaluate the MIC value of FLC of the SN152 strain in YPD medium 1 μg/mL CZ66, 1 μg/mL geldanamycin, or 1 μg/mL geldanamycin and 1 μg/mL CZ66 or without any compound (as control) (Left). Cells from the broth microdilution assays were spotted onto YPD medium and incubated at 30°C for 48 h before the plate was photographed (Right). Download FIG S2, TIF file, 0.2 MB. Copyright © 2022 Lu et al. 2022 Lu et al. https://creativecommons.org/licenses/by/4.0/ This content is distributed under the terms of the Creative Commons Attribution 4.0 International license.

CZ66 eliminated the FLC tolerance of C. albicans and slightly increased the MIC value of FLC (Fig. 1C). Reasons for such an increase in the MIC values of antifungal agents could include (i) increased expression of the target proteins of antifungal agents and (ii) activities of drug efflux pump proteins (36). There is evidence that the efflux pumps are not involved in *C. albican*’s FLC tolerance (6) and CZ66 did not affect the efflux activity of Cdr1 as assayed by rhodamine 6G efflux (Fig. S2A). Therefore, CZ66 may slightly elevate the MIC value of FLC by activating the expression of ergosterol synthesis-related genes. Indeed, our RNA-seq analysis showed that FLC combined with CZ66 induced the expression of the *ERG11* gene compared with FLC alone (4 μg/mL FLC plus 1 μg/mL CZ66 versus 4 μg/mL FLC) (Table S3), suggesting that CZ66 enhances FLC depletion of intracellular ergosterol in C. albicans.

10.1128/mbio.02639-22.10 TABLE S3 Differential expression of genes between *C. albicans* cells treated with FLC plus CZ66 and cells treated with FLC alone. Download Table S3, XLSX file, 0.02 MB. Copyright © 2022 Lu et al. 2022 Lu et al. https://creativecommons.org/licenses/by/4.0/ This content is distributed under the terms of the Creative Commons Attribution 4.0 International license.

Since CZ66 has an enhanced inhibitory effect of FLC on ergosterol biosynthesis, we hypothesized that an increase in the cellular ergosterol content might neutralize the synthetic lethality of CZ66 and FLC. To test this hypothesis, we examined the effect of feeding exogenous ergosterol on the susceptibility of isolate SC5314 to FLC and CZ66. Broth microdilution assays indicated that 100 μM ergosterol counteracted the synergistic lethal effect of CZ66 and FLC, supporting our hypothesis that the synthetic lethality of CZ66 and FLC can be abolished in the presence of exogenous ergosterol (Fig. 3B and C).

Calcineurin plays an essential role in the FLC tolerance of C. albicans (8), and Hsp90 contributes to FLC tolerance by stabilizing calcineurin (9, 17, 37). Therefore, inhibitors of calcineurin (cyclosporine) and Hsp90 (geldanamycin) can make FLC fungicidal (9, 15) (Fig. S2B). When CZ66 is combined with cyclosporine or geldanamycin, the synergistic fungicidal effect of cyclosporine or geldanamycin with FLC can be masked and CZ66 can slightly increase the MIC value of FLC (Fig. S2C). In addition, the Hsp90-calcineurin signaling pathway governs resistance to caspofungin and terbinafine (38–40) in C. albicans. However, CZ66 did not enhance the antifungal effects of these agents (Fig. 2D and Fig. S1C). These results suggest that the synergistic lethality of CZ66 and FLC is independent of the Hsp90-calcineurin signaling pathway.

### Erg251 is a potential target of CZ66.

**FIG 4:**
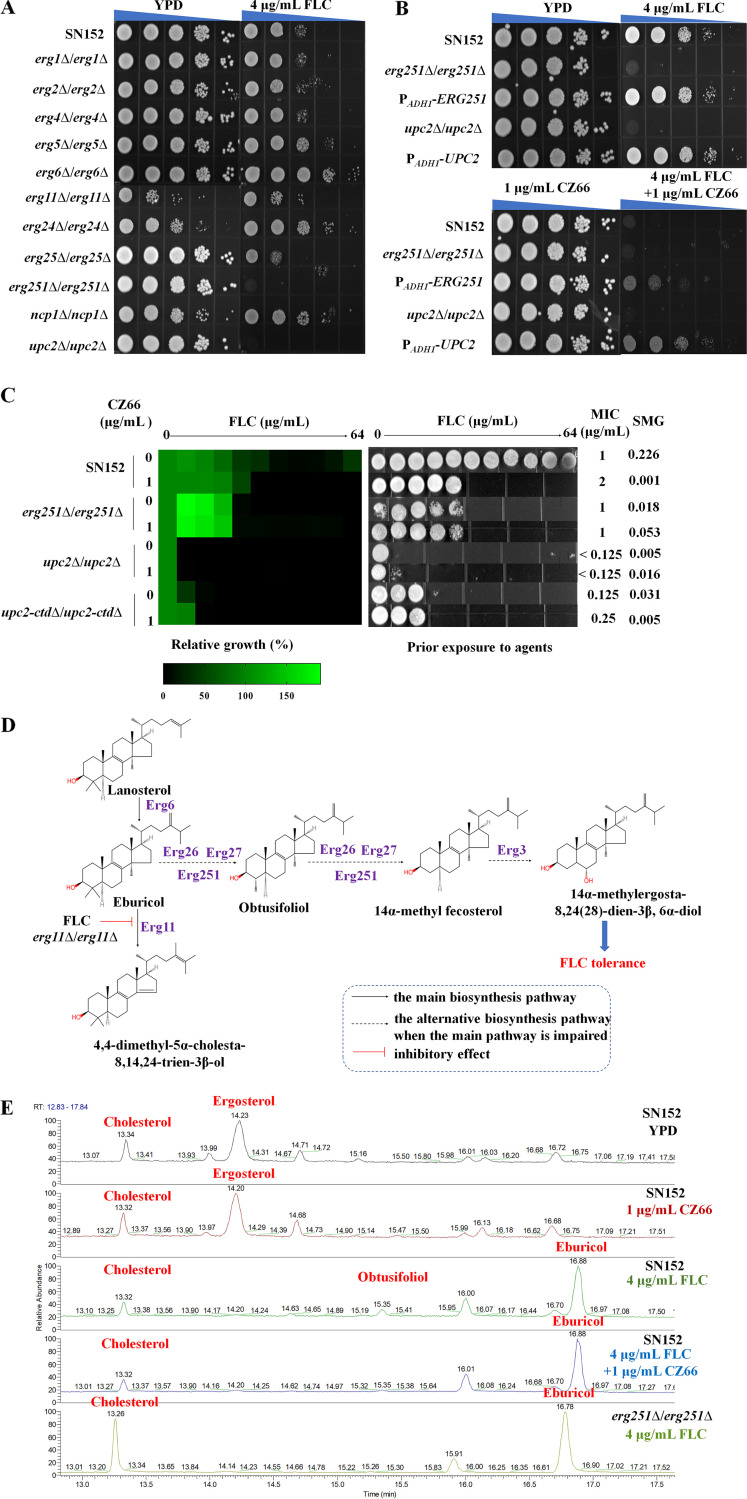
Erg251 is a potential target of CZ66. (A and B) Overnight C. albicans cultures were adjusted to 1 × 10^7^ cells/mL and then were spotted (1:10 dilution) onto solid YPD medium with 4 μg/mL FLC, 1 μg/mL CZ66, or 4 μg/mL FLC and 1 μg/mL CZ66 or without any compound and photographed after 48 h of incubation at 30°C. (C) The broth microdilution assays showed that the *erg251*Δ/*erg251*Δ null mutant had an identical FLC MIC value to the SN152 strain in the presence of CZ66 (1 μg/mL) incubated in YPD medium at 30°C for 48 h. Cells from the broth microdilution assays were spotted onto YPD medium and incubated at 30°C for 48 h before the plate was photographed. (D) A schematic diagram of an alternative sterol synthetic pathway was constructed by C-4 sterol methyl oxidase (Erg251), C-3 sterol dehydrogenase (Erg26), 3-keto sterol reductase (Erg27), and C-5 sterol desaturase (Erg3). (E) Quantification of the percentage of ergosterol in C. albicans after treatment of the SN152 strain in the following way for 12 h: (i) 0.1% DMSO (as a control group), (ii) 4 μg/mL FLC, (iii) 1 μg/mL CZ66, and (iv) 4 μg/mL FLC plus 1 μg/mL CZ66 and exposing the *erg251*Δ/*erg251*Δ mutant strain to 4 μg/mL FLC.

Because CZ66 has suppressive effects on ergosterol synthesis, we hypothesized that a potential target protein of CZ66 is involved in the ergosterol synthesis pathway. We constructed null mutants of ergosterol synthesis-related genes that are regulated by the transcription factor Upc2 (41), generating *erg1*Δ/*erg1*Δ, *erg2*Δ/*erg2*Δ, *erg4*Δ/*erg4*Δ, *erg5*Δ/*erg5*Δ, *erg6*Δ/*erg6*Δ, *erg11*Δ/*erg11*Δ, *erg24*Δ/*erg24*Δ, *erg25*Δ/*erg25*Δ, *erg251*Δ/*erg251*Δ, *ncp1*Δ/*ncp1*Δ, and *upc2*Δ/*upc2*Δ null mutants. We failed to construct homozygous deletion strains for the *ERG9* and *ERG10* genes, suggesting that the *ERG9* and *ERG10* genes are essential for C. albicans survival (42, 43) and indicating that they are not potential targets of CZ66 as CZ66 has no inhibitory effect on the growth of C. albicans (Fig. 1C and E). Spot assay results showed that the *erg251*Δ/*erg251*Δ and *upc2*Δ/*upc2*Δ null mutants are hypersensitive to FLC (Fig. 4A), similar to wild-type C. albicans exposed to FLC and CZ66 (Fig. 2A) and consistent with previous reports (44–47). There was no significant difference in the sensitivities of the *erg251*Δ/*erg251*Δ and *upc2*Δ/*upc2*Δ null mutants to caspofungin, amphotericin B, and 5-fluorocytosine (Fig. S3A), which is consistent with CZ66 failing to enhance the antifungal activities of these agents (Fig. 2E and Fig. S1E and I). We then created ectopic overexpression constructs of the *ERG251* and *UPC2* genes in C. albicans strain SN152 by expressing either the *ERG251* gene or the *UPC2* gene using the potent *ADH1* promoter (48). Spot assay results showed that in C. albicans, ectopic overexpression of either *ERG251* or *UPC2* counteracted the synergistic fungicidal effect of FLC and CZ66 (Fig. 4B). These results suggested that Erg251 and Upc2 are potential targets of CZ66.

10.1128/mbio.02639-22.4 FIG S3 Overnight C. albicans culture was adjusted to 1 × 10^7^ cells/mL and then was spotted (1:10 dilution) onto solid YPD medium or containing the indicated compounds and photographed after 48 h of incubation at 30°C. Download FIG S3, TIF file, 0.7 MB. Copyright © 2022 Lu et al. 2022 Lu et al. https://creativecommons.org/licenses/by/4.0/ This content is distributed under the terms of the Creative Commons Attribution 4.0 International license.

In C. albicans, Upc2 not only contributes to FLC tolerance but also contributes to FLC resistance (45). Deletion of the C-terminal activation loop of Upc2 generated at least 30% lower transcriptional activity that did not increase on FLC treatment relative to the uninduced wild type (49), suggesting that the C-terminal activation loop of Upc2 may only be involved in FLC tolerance, but not in FLC resistance. We constructed a homozygous deletion of the C-terminal 75 residues (Δ1912–2139) of Upc2 (*upc2*-*ctd*Δ/*upc2*-*ctd*Δ), and broth microdilution assays showed that the *upc2*Δ/*upc2*Δ and the *upc2*-*ctd*Δ/*upc2*-*ctd*Δ mutants not only abolished FLC tolerance of C. albicans but also decreased the MIC values of FLC (Fig. 4C). However, CZ66 only eliminated the FLC tolerance of C. albicans and did not reduce the MIC value of FLC (Fig. 1C and D). This paradoxical phenotype suggests that Upc2 is unlikely to be a target of CZ66. In contrast, the *erg251*Δ/*erg251*Δ null mutant displayed the identical FLC MIC value to the wild-type strain SN152 in the presence of CZ66, and the FLC susceptibility of the *erg251*Δ/*erg251*Δ null mutant was the same whether it was exposed to CZ66 or not (Fig. 4C). The pattern of the FLC sensitivity phenotype suggested that Erg251 is a potential target of CZ66. Because Upc2 is a transcriptional regulator of Erg251 (41), both *erg251*Δ/*erg251*Δ and *upc2*Δ/*upc2*Δ mutants exhibit similar phenotypes when exposed to FLC.

When FLC inhibits the activity of Erg11 and impairs the main ergosterol synthetic pathway, an alternative sterol synthetic pathway involving C-4 sterol methyl oxidase (Erg251), C-3 sterol dehydrogenase (Erg26), 3-keto sterol reductase (Erg27), and C-5 sterol desaturase (Erg3) produces the replacement sterols obtusifoliol, 14α-methylfecosterol, and 14α-methylergosta-8,24(28)-dien-3β, 6α-diol (11, 14) (Fig. 4D). If CZ66 targets Erg251 and inhibits its activity, when FLC inhibits the Erg11 activity, CZ66 should inhibit obtusifoliol synthesis. To test this conjecture, we quantified the percentage of ergosterol in C. albicans after treating the samples in the following way for 12 h: (i) 0.1% dimethyl sulfoxide (DMSO) (as a control group), (ii) 4 μg/mL FLC, (iii) 1 μg/mL CZ66, and (iv) 4 μg/mL FLC plus 1 μg/mL CZ66. Our results showed that FLC inhibited ergosterol synthesis and increased the levels of eburicol and obtusifoliol. When FLC was combined with CZ66, the ergosterol content of C. albicans decreased, and the amount of eburicol increased, but the amount of obtusifoliol did not increase (Fig. 4E). This change in sterol composition caused by FLC plus CZ66 resembles the altered sterol composition seen when the *erg251*Δ/*erg251*Δ null mutant is exposed to FLC (Fig. 4E). These results further suggest that CZ66 inhibits the activity of Erg251 and prevents C. albicans from using the alternative sterol synthetic pathway that synthesizes 14α-methylsterols.

### Erg251 plays a vital role in 14α-methylsterol synthesis.

We ectopically overexpressed the *ERG251*, *ERG25*, *ERG26*, and *ERG27* genes in strain SN152. We found that strains with ectopic overexpression of any of these four genes displayed increased resistance to FLC (Fig. 4B and Fig. S3B), suggesting that increased synthesis of 14α-methylsterols can augment the resistance of C. albicans to FLC. We further constructed null mutants of the 14α-methylsterol synthesis-related genes, creating *erg3*Δ/*erg3*Δ, *erg26*Δ/*erg26*Δ, and *erg27*Δ/*erg27*Δ null mutants. Only the *erg251*Δ/*erg251*Δ null mutant fails to grow in the presence of FLC (Fig. 4A to C). The homozygous deletion of the *ERG3* gene resulted in a decreased sensitivity to FLC (Fig. S3C) (50). In contrast, the loss of the *ERG25* and *ERG26* genes increased sensitivity to FLC, but the *erg25*Δ/*erg25*Δ and *erg26*Δ/*erg26Δ* null mutants still can grow when exposed to FLC (Fig. 4A and Fig. S3C), while the lack of the *ERG27* gene seems not to affect the sensitivity of C. albicans to FLC (Fig. S3C). Loss of Erg3 resulted in increased FLC resistance of C. albicans due to the depletion of 14α-methyl-3-6-diol produced by Erg3 (the Δ-5,6-desaturase) (50). It is worth noting that CZ66, but not cyclosporine (50), can eliminate FLC resistance of the *erg3*Δ/*erg3*Δ null mutant (Fig. S3C), indicating that inhibition of Erg251 activity counteracted the 14α-methyl-3-6-diol deficiency-induced increased FLC resistance.

**FIG 5:**
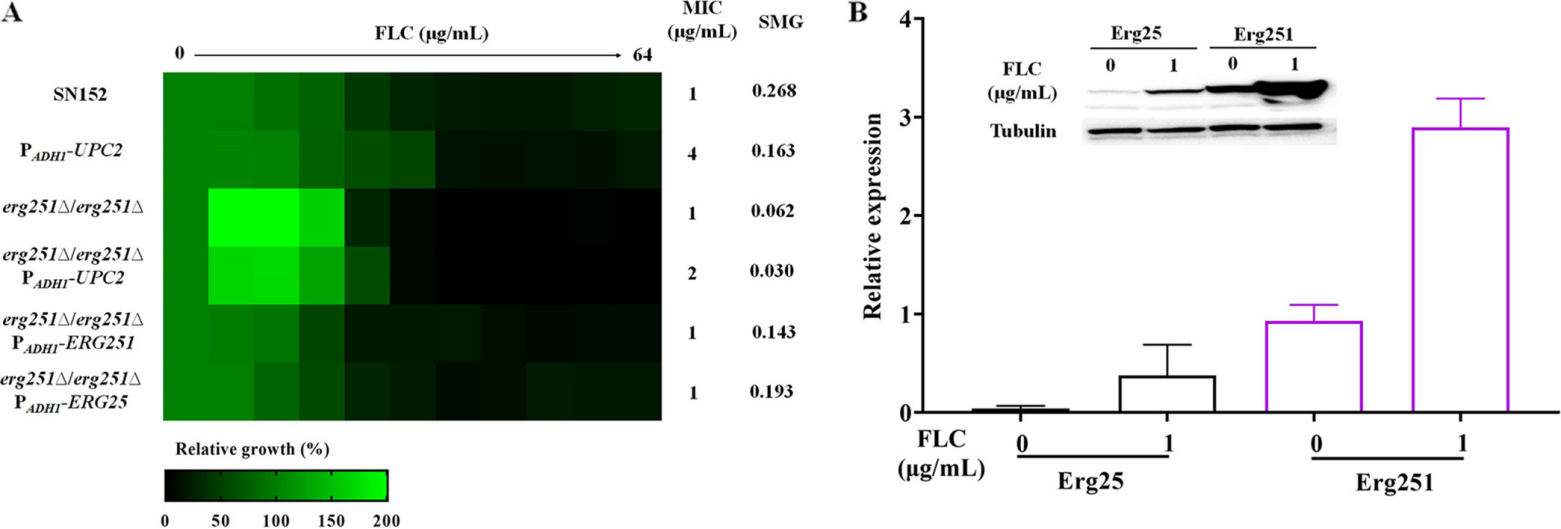
Erg251 is important for 14α-methylsterol synthesis. (A) Broth microdilution assays (incubated in YPD medium at 30°C for 48 h) were used to determine the FLC susceptibility of the SN152, ADH1p-*UPC2*, *erg251*Δ/*erg251*Δ, *erg251*Δ/*erg251*Δ::ADH1p-*UPC2*, *erg251*Δ/*erg251*Δ::ADH1p-*ERG251*, and *erg251*Δ/*erg251*Δ::ADH1p-*ERG25* strains. (B) The expression levels of Erg25 and Erg251 in the presence (1 μg/mL) or absence of FLC were detected by immunoblotting.

We further engineered ectopic overexpression of the *UPC2* gene in the *erg251Δ/erg251Δ* null mutant by expressing the *UPC2* gene using the strong *ADH1* promoter (*erg251Δ/erg251Δ*::ADH1p-*UPC2*) (48). We found that for the *erg251Δ/erg251Δ*::ADH1p-*UPC2* mutant, the MIC value of FLC was slightly elevated compared to that of the *erg251Δ/erg251Δ* null mutant, but the overexpression mutant still did not display FLC tolerance (Fig. 5A). These results suggested that Erg251, a C-4 sterol methyl oxidase, plays an essential role in the 14α-methylsterol synthesis and FLC tolerance in C. albicans.

Erg25 is a paralog to Erg251 and is proposed to be also a C-4 methylsterol oxidase. Nevertheless, we found that Erg251 plays a more important role in FLC tolerance in C. albicans because while the *erg251*Δ/*erg251*Δ strain was inviable in the presence of FLC, the *erg25*Δ/*erg25*Δ strain remained viable in its presence (Fig. 4A). Further, we found that ectopic overexpression of the *ERG25* gene (regulated by the *ADH1* promoter) in the *erg251*Δ/*erg251*Δ null mutant could compensate for the loss of FLC tolerance (Fig. 5A). Therefore, we conjecture that Erg25 does not contribute to FLC tolerance of C. albicans, maybe because its expression level is much lower than that of Erg251. To verify this hypothesis, we tagged the C termini of Erg25 and Erg251 with green fluorescent protein (GFP) (48). We then cultured the Erg25-GFP mutant and the Erg251-GFP mutant in the presence (1 μg/mL) or absence of FLC. We tested the expression of Erg25 and Erg251 using an anti-GFP antibody and found that the basal expression level of Erg251 was much higher than that of Erg25 in the absence of FLC (Fig. 5B). Furthermore, FLC can induce the expression of Erg251 significantly more than Erg25 (Fig. 5B). Therefore, we believe that the expression level causes the difference in the function of the two genes in FLC tolerance and C-4 methylsterol oxidase function is mainly encoded by the *ERG251* gene in C. albicans.

**FIG 6:**
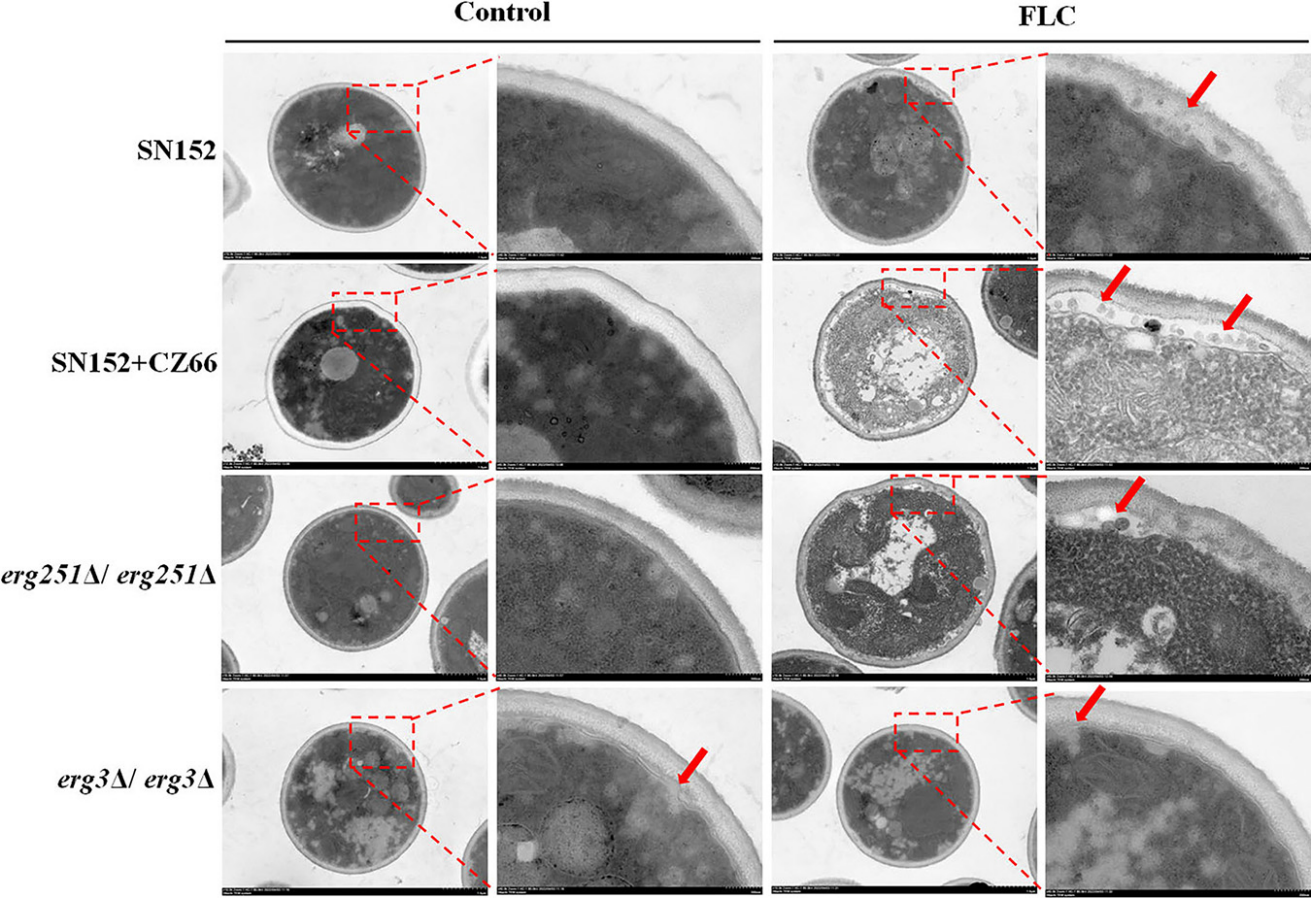
Integrity of cell membrane by the *ERG251* gene deletion or CZ66. Shown are ultrastructure images of C. albicans SN152, SN152 plus 1 μg/mL CZ66, the *erg251*Δ/*erg251*Δ strain, and the *erg3*Δ/*erg3*Δ strain in the presence or absence of FLC (4 μg/mL) for 12 h. Red arrows indicate the damage to the membranes.

These abnormal sterols with the 14α-methyl group exert membrane stress when incorporated into the fungal membrane and ultimately inhibit proliferation (12–14, 51), but C. albicans cells can still survive in the presence of azoles (3) due to 14α-methylsterols substituting for ergosterol, resulting in maintained C. albicans cell membrane integrity. We determined the ultrastructure of membranes of strain SN152, SN152 plus 1 μg/mL CZ66, *erg251*Δ/*erg251*Δ, and *erg3*Δ/*erg3*Δ strains with or without the action of FLC (4 μg/mL for 12 h) via transmission electron microscopy. The deletion of the *ERG251* gene or 1 μg/mL CZ66 resulted in little damage to the plasma membrane of C. albicans, but in the presence of 4 μg/mL FLC, the cell membrane of the *erg251*Δ/*erg251*Δ strain and strain SN152 plus 1 μg/mL CZ66 shriveled dramatically, and a large amount of entocyte leaked into the gap of the cell membrane and the cell wall (Fig. 6). Although the deletion of the *ERG3* gene resulted in slight damage to the plasma membrane of C. albicans, 4 μg/mL FLC did not aggravate the damage to the cell membrane in the *erg3*Δ/*erg3*Δ strain (Fig. 6). Therefore, Erg251 plays a crucial role in cell membrane integrity when C. albicans is exposed to FLC.

## DISCUSSION

FLC tolerance contributes to the emergence of fungal FLC resistance and the recurrence of fungal infections (3, 6). Therefore, blocking FLC tolerance and making FLC fungicidal will be important for treating invasive fungal infections. Combinations of FLC and adjuvants are a promising strategy to make FLC fungicidal and boost the antifungal capacity of FLC (2, 52). Various adjuvants have been identified that can turn FLC fungicidal through different mechanisms. Inhibition of the Hsp90-calcineurin signaling pathway can eliminate the FLC tolerance of C. albicans. This can be accomplished by inhibitors of calmodulin (fluphenazine, chlorpromazine, and tamoxifen) (6, 53, 54) (see Fig. S4A in the supplemental material), calcineurin (cyclosporine and tacrolimus) (8, 55), and Hsp90 (geldanamycin and radicicol) (9). In addition, disruptions of the cell wall and membrane integrities (2, 15), inhibiting ADP ribosylation factor activities (10), depletion of intracellular ergosterol and sphingolipids (25, 26, 32, 33) (Fig. S4B to D), and stresses by hyperosmotic pressure (2), temperature (56), and pH (57) also can abolish FLC tolerance of C. albicans. In this study, we found a novel inhibitor of Erg251, named CZ66 (Fig. 1A), which made FLC fungicidal by inhibiting the synthesis of 14α-methylsterols.

10.1128/mbio.02639-22.5 FIG S4 Broth microdilution assays (incubated in YPD medium at 30°C for 48 h) were used to evaluate the synergistic effect of FLC and antifungal agents, including (A) tamoxifen, (B) cerulenin, (C) rapamycin, and (D) myriocin. Download FIG S4, TIF file, 0.1 MB. Copyright © 2022 Lu et al. 2022 Lu et al. https://creativecommons.org/licenses/by/4.0/ This content is distributed under the terms of the Creative Commons Attribution 4.0 International license.

**FIG 7:**
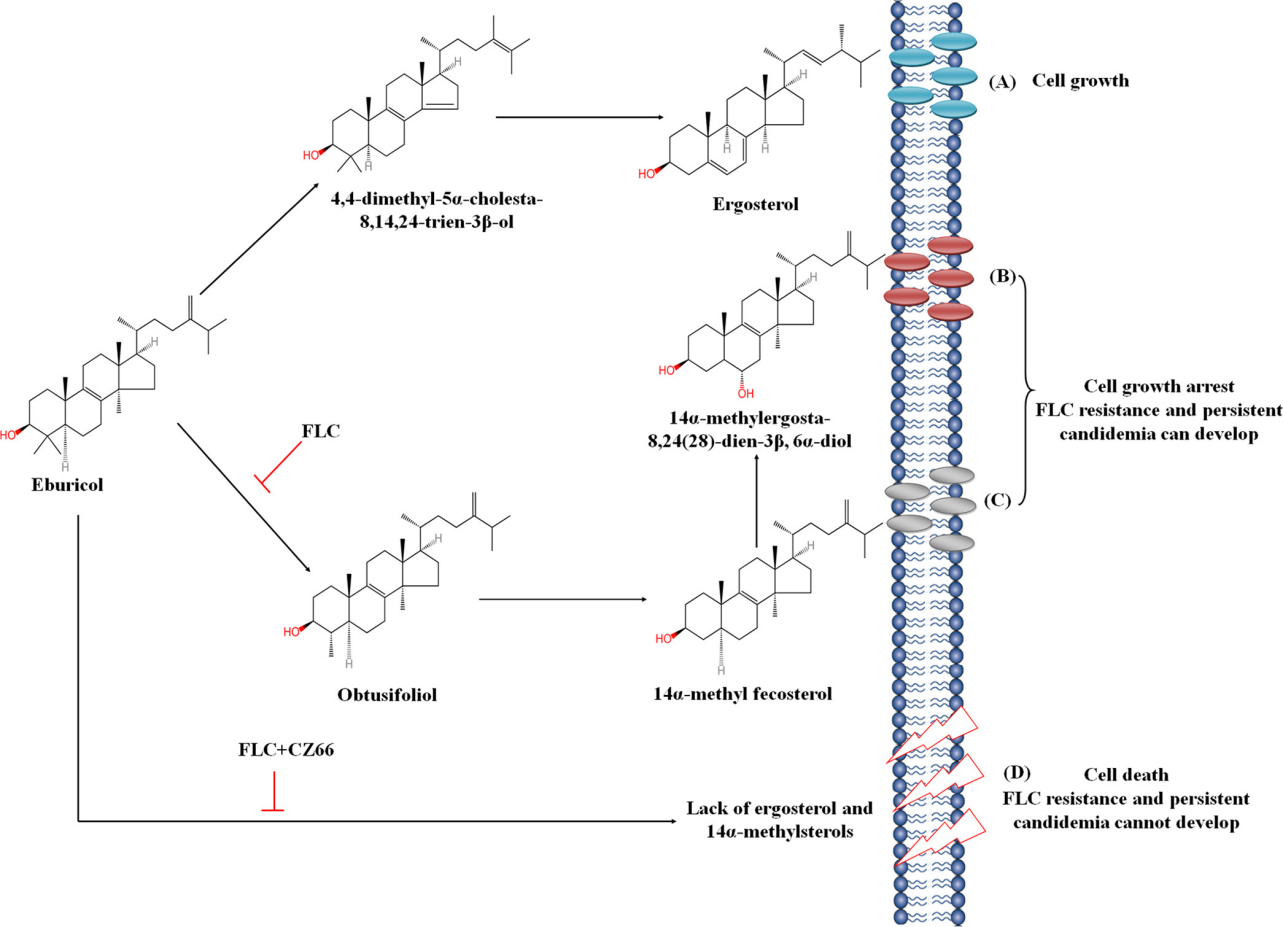
Schematic diagram of a mechanism of CZ66 eliminating FLC tolerance of C. albicans. (A) Ergosterol is important for the cell membrane integrity of *C. albcians*. (B) FLC targets the C14α-demethylase (Erg11) and consequently results in ergosterol depletion and accumulation of 14α-methylsterols, such as 14α-methyl-3-6-diol. These 14α-methylsterols replace ergosterol, resulting in a functionally maintained C. albicans cell membrane and making C. albicans survive in the presence of FLC. (C) The deletion of the *ERG3* gene leads to the loss of 14α-methyl-3-6-diol, but accumulated 14α-methylfecosterol is sufficient to maintain C. albicans cell membrane functionally. (D) The *ERG251* gene deletion or CZ66 inhibits the activity of Erg251 and obtusifoliol will not be produced for the synthesis of 14α-methylsterols. This leads to the absence of 14α-methylsterols as a substitute for ergosterol to maintain cell membrane integrity and, consequently, makes C. albicans inviable in the presence of FLC.

Azoles are directed against the C14α-demethylase in the ergosterol pathway and consequently result in ergosterol depletion and accumulation of 14α-methylsterols, such as 14α-methyl-3-6-diol (11). These abnormal sterols with the 14α-methyl group exert membrane stress when incorporated into the fungal membrane and ultimately inhibit proliferation (12–14, 51). However, C. albicans cells can still survive in the presence of azoles (3) due to substituting 14α-methylsterols for ergosterol, resulting in a functionally maintained C. albicans cell membrane. Therefore, 14α-methylsterols can play a dual role in the antifungal effect of azoles in that they enhance the antifungal effect of azoles and trigger C. albicans growth arrest but provide C. albicans the ability to tolerate azoles. Therefore, we believe inhibiting the production of 14α-methylsterols can eliminate the azole tolerance of *C. albicans*. However, the longstanding idea is that increasing the content of toxic 14α-methylsterols improves the antifungal activity of azoles (51). Indeed, the homozygous deletion of the *ERG3* gene resulted in decreased sensitivity to FLC (Fig. S3B) (50) because of the depletion of 14α-methyl-3-6-diol produced by Erg3 (the Δ-5,6-desaturase). Here, we found that when the *ERG251* gene deletion or CZ66 inhibits the activity of Erg251, obtusifoliol will not be produced for the synthesis of 14α-methylsterols, such as 14α-methyl-3-6-diol. This leads to the absence of 14α-methylsterols as a substitute for ergosterol to maintain cell membrane integrity and, consequently, makes C. albicans inviable in the presence of azoles (Fig. 7). It is worth noting that unlike the Hsp90-calcineurin pathway, which affects the sensitivity of C. albicans to various antifungal drugs (9, 15, 38–40), inhibiting the synthesis of 14α-methylsterols will specifically enhance the susceptibility of C. albicans to azoles. Hence, targeting Erg251 (such as by CZ66) and inhibiting the synthesis of 14α-methylsterols provide a new strategy for developing FLC adjuvants to make FLC fungicidal.

We modeled the interaction between Erg251 and CZ66 using molecular docking and molecular dynamics (MD) simulations (Fig. S5A and see details in Text S1). We found that CZ66 was most likely to interact with Erg251 with Glu195, Gly206, and Arg241 in the complex at the simulation of 6 to 10 ns (Fig. S5B and C). His183 and Gly206 formed aromatic hydrogen bonds in the complex, and Arg241 and Glu195 formed hydrogen bonds with CZ66 (Fig. S5B). If these three amino acids of Erg251 play an important role in the interaction between Erg251 and CZ66, their mutation will block the killing by CZ66 in the presence of FLC. Therefore, we mutated these three residues (Glu195Ala, Gly206Ala, and Arg241Ala) of Erg251 and introduced the mutated Erg251 into the *erg251*Δ/*erg251*Δ mutant. However, the mutated Erg251 cannot neutralize the increased FLC susceptibility of the *erg251*Δ/*erg251*Δ mutant, suggesting these three residues (Glu195, Gly206, and Arg241) are essential for the activity of Erg251. We hope to confirm the interaction between CZ66 and Erg251 by surface plasmon resonance analysis, but we have not successfully expressed Erg251, a membrane protein with low solubility, in Escherichia coli.

10.1128/mbio.02639-22.1 TEXT S1 Erg251 and CZ66 interaction by molecular docking and molecular dynamics simulation. Download Text S1, DOCX file, 1.5 MB. Copyright © 2022 Lu et al. 2022 Lu et al. https://creativecommons.org/licenses/by/4.0/ This content is distributed under the terms of the Creative Commons Attribution 4.0 International license.

10.1128/mbio.02639-22.6 FIG S5 Representative binding poses between Erg251 and CZ66 during the simulation. (A) CZ66 (green) was located in the binding area of Erg251. (B) Two-dimensional interaction diagram between CZ66 and the residues of Erg251. (C) CZ66 interacted with the residues in the interface of Erg251. Download FIG S5, JPG file, 0.5 MB. Copyright © 2022 Lu et al. 2022 Lu et al. https://creativecommons.org/licenses/by/4.0/ This content is distributed under the terms of the Creative Commons Attribution 4.0 International license.

It is worth noting that Erg251 has a low level of identity (<40%) (58) to human proteins, suggesting compounds targeting Erg251 would have low toxicity. We found that CZ66 has low toxicity to human umbilical vein endothelial cells (HUVECs) (Fig. S6A), indicating that CZ66 can be a potential alternative to geldanamycin and cyclosporine as an FLC adjuvant. However, we did not reproduce the potent *in vitro* synergy of FLC and CZ66 in animal models because CZ66 failed to enhance the antifungal activity of FLC *in vivo* (Fig. S6B), potentially due to CZ66 instability *in vivo* (data not shown).

10.1128/mbio.02639-22.7 FIG S6 (A) CZ66 had low toxicity to human umbilical vein endothelial cells (ATCC 1730). Even when the concentration of CZ66 was as high as 32 μg/mL, it still hardly inhibited the growth of cells. (B) Female BALB/C mice (6 to 8 weeks old) were infected by SN152, treated with 1 mg/kg FLC or 1 mg/kg FLC plus 1 mg/kg CZ66 once a day intraperitoneally for 3 days, and observed for 31 days. Each group contains eight mice. Download FIG S6, TIF file, 0.1 MB. Copyright © 2022 Lu et al. 2022 Lu et al. https://creativecommons.org/licenses/by/4.0/ This content is distributed under the terms of the Creative Commons Attribution 4.0 International license.

In conclusion, in the present study, we found that CZ66 makes FLC fungicidal by targeting Erg251. Erg251 is a synthetic lethal target protein of FLC because the loss of Erg251 results in the depletion of 14α-methyl group sterols, and these sterols contribute to FLC tolerance in C. albicans. Our findings provide the molecular basis for directly identifying specific small molecule inhibitors of Erg251 and open the way for abolishing fungal FLC tolerance by inhibiting 14α-methyl-group-sterol synthesis.

## MATERIALS AND METHODS

### Strains, primers, agents, and cultural conditions.

All strains and primers used in this study are listed in Table S1 and Table S2 in the supplemental material. *Candida* strains were routinely cultured at 30°C in YPD medium (1% yeast extract, 2% peptone, 2% dextrose). To test whether the antifungal activity of CZ66 depends on the carbon source, we used the following media: YP (1% yeast extract and 2% peptone), YPFructose (YP medium plus 2% fructose), YPGalactose (YP medium plus 2% galactose), YPGlcNAc (YP medium plus 2% *N*-acetyl-d-glucosamine [GlcNAc]), YPMannose (YP medium plus 2% mannose), YPSucrose (YP medium plus 2% sucrose), YPGlycerol (YP medium plus 2% glycerol), and YPEthanol (YP medium plus 2% ethanol). To delete target genes, we used a synthetic medium (2% dextrose, 6.7% yeast nitrogen base without amino acids). Plates containing medium were supplemented with 2% agar. Drug stock solutions were prepared using dimethyl sulfoxide (DMSO) (Sangon Biotech, Shanghai, China) as a solvent for FLC (6.4 mg/mL) (Aladdin, Shanghai, China), CZ66 (6.4 mg/mL) (20), cerulenin (6.4 mg/mL) (Aladdin, Shanghai, China), fluvastatin (6.4 mg/mL) (Aladdin, Shanghai, China), terbinafine (6.4 mg/mL) (Aladdin, Shanghai, China), amphotericin B (6.4 mg/mL) (Aladdin, Shanghai, China), Congo red (6.4 mg/mL) (Sangon Biotech, Shanghai, China), 5-fluorocytosine (Aladdin, Shanghai, China), and myriocin (Aladdin, Shanghai, China), using sterilized deionized water as a solvent for SDS (10%) (Sangon Biotech, Shanghai, China) and using 50% Tween 80–50% ethanol as a solvent for ergosterol (10 mM) (Sangon Biotech, Shanghai, China).

10.1128/mbio.02639-22.8 TABLE S1 Strains used in this study. Download Table S1, XLSX file, 0.01 MB. Copyright © 2022 Lu et al. 2022 Lu et al. https://creativecommons.org/licenses/by/4.0/ This content is distributed under the terms of the Creative Commons Attribution 4.0 International license.

10.1128/mbio.02639-22.9 TABLE S2 Primers used in this study. Download Table S2, XLSX file, 0.02 MB. Copyright © 2022 Lu et al. 2022 Lu et al. https://creativecommons.org/licenses/by/4.0/ This content is distributed under the terms of the Creative Commons Attribution 4.0 International license.

### Antifungal susceptibility testing.

Initial antifungal sensitivity testing was done using a modified version of (CLSI procedure M27, 4th ed. [https://www.clsi.org/]). Briefly, 100 μL of drugs at 2-fold the final concentration was serially diluted in flat-bottom 96-well plates and combined with 100 μL of overnight C. albicans cultures adjusted to 1 × 10^3^ cells/mL. The plates were incubated at 30°C without shaking, and optical densities (ODs) were read with a Thermo Scientific plate reader at 48 h. The MIC was determined by the first well with a growth reduction of >50% in terms of OD_595_ values in the presence of a compound compared to untreated control cells.

Dose-matrix titration assays were used to evaluate drug synergies (10). Briefly, dose-matrix titration assays were done as described for the MIC assays. Fifty microliters of 4-fold the final drug concentration of drug A was dispensed in 2-fold serial dilution steps across seven plate columns, and then, 50 μL of 4-fold the final drug concentration of drug B was dispensed in 2-fold serial dilution steps down seven rows of the plate. One hundred microliters of overnight C. albicans cultures adjusted to 1 × 10^3^ cells/mL was dispensed in all drug-containing wells plus one control well containing no drugs.

Before the 48-h OD_595_ readings, plates were carefully shaken so that a representative aliquot of 2 μL of each well could be spotted on fresh YPD recovery plates to assess the extent to which cells recover from the drug treatments. Recovery plates were incubated at 30°C for 48 h before being photographed. All assays were performed and repeated three times.

### Spotting assay.

The spotting assay was performed as described previously (59). Briefly, after growth overnight, mid-log-phase cultures of strains required for experimental conditions were adjusted to 1 × 10^7^ cells/mL using a hemacytometer, and then 1:10 serially diluted in sterile phosphate-buffered saline (PBS) and spotted onto indicated plates with an indicated concentration of compounds. After 48 h of incubation at 30°C, pictures of the growth of cells were taken. Assays were repeated three times.

### Disk diffusion assay.

The CLSI M44-A2 guidelines for antifungal disk diffusion susceptibility testing were followed with slight modifications. Strains were grown on agar plates, and cell density was adjusted to 1 × 10^6^ cells/mL, as described above. One hundred microliters of cell suspension was streaked onto plates. One paper disk (Liofilchem, Italy) supplemented with 25 μg FLC was placed in the center of each plate. The plates were then incubated for 48 h and photographed. Assays were performed three times.

### Growth inhibition curve assays.

Growth rate determination was performed as described previously (59). C. albicans strain SC5314 was grown in YPD medium overnight on a rotary shaker (200 rpm) at 30°C. The overnight culture was inoculated into 100 mL of fresh YPD medium to a final optical density of 0.05 at 595 nm. C. albicans cells were divided into four groups and treated with the following agents: (i) 0.1% DMSO (as a control group), (ii) 4 μg/mL FLC, (iii) 1 μg/mL CZ66, and (iv) 4 μg/mL FLC plus 1 μg/mL CZ66. The optical density was measured every 2 h until the stationary phase of the growth curve was reached. Assays were performed in duplicate three times.

### Time-kill curve assays.

For the time-kill curve assays, overnight C. albicans cultures were diluted to achieve final cell densities of 10^5^ cells/mL in 100 mL of fresh YPD medium. Agents were added to the cultures to achieve final concentrations of 0.1% DMSO (as a control group), 4 μg/mL FLC, 1 μg/mL CZ66, and 4 μg/mL FLC plus 1 μg/mL CZ66. Following every 2 h of exposure, triplicate-sample aliquots were removed, serially diluted, and plated on an appropriate drug-free agar medium. After incubation of 48 h at 30°C viable colonies were enumerated. The rate and extent of the fungal killing were determined by plotting the reduction in viable colony counts (log_10_ cells per milliliter) against time.

### Disruption of ergosterol synthesis-related genes.

The two alleles of the genes of interest were deleted from strain SN152 using fusion PCR (60). The first round of reactions involved the amplification of the flanking sequences of target genes (with a template of genomic DNA and primers P1 and P3 or P4 and P6, in separate reactions) and the selectable marker (*HIS1* or *ARG4*) (with a template of plasmid pSN52 or pSN69 and primers universal primer 2 and universal primer 5). The 5′ tails of universal primer 2 and P3 are complementary, as are the 5′ tails of P4 and universal primer 5. All three first-round products were combined, and the fusion product was amplified with primers P1 and P6. The transformation was carried out according to the protocols of the Yeastmaker yeast transformation system 2 kit (Clontech Laboratories, Inc.) and selected on synthetic medium containing the necessary auxotrophic supplements for heterozygous mutant strains and on synthetic medium containing the necessary auxotrophic supplements and 100 μM ergosterol for homozygous null mutant strains. The following primers were used for diagnosis of target gene knockouts: for the 5′ junctions, the primers were UCheck plus primer HIS1left or ARG4left, as appropriate; for the 3′ junctions, the primers were Dcheck plus HIS1right or ARG4right.

### Ectopic overexpression of target genes.

As described previously, strains of ectopically overexpressed target genes were constructed (48). The target genes were first introduced into the pCPC18 vector for ectopic overexpression. The first round of PCR used F1/R1 primers to amplify the target gene, generating a product with a 15-bp flank homology region. The pCPC18 backbone was amplified using primers CaP19 and CaP28. Then these two products were assembled via ligation-independent cloning (61). Verification primers VP18 and VP26 were used to check the insertion of the target gene. The second round of PCR used primers CaP22 and CaP23 to generate the ectopic expression cassette. After transformation and integration, the target gene was integrated into the *ADE2* locus and controlled by the constitutive *ADH1* promoter. Verification primers VP28 and VP29 were used for checking the 5′ integration, and VP30 and VP31 were used for the 3′ integration.

### C-terminal tagging of proteins with GFP.

To tag the C-terminal ends of Erg25 and Erg251 using GFP, we adopted a PCR strategy to amplify the desired DNA cassettes in plasmid pCPC64 (48). The F1 primer comprises a 20-bp homology to GFP and a 39-bp sequence precisely before the stop codon of the target gene. For the first round of PCR using F1/R1 primers, a product with 39-bp homology regions was generated. Using this product as a PCR template directly, the second round of PCR using F2/R2 primers yielded DNA cassettes with 78-bp homology regions to the target gene. This product could be transformed into C. albicans cells to generate a strain with the C-terminal-tagged gene. We used primers target gene check-F and VP8 to check the 5′ integration and target gene check-R and VP19 for the 3′ integration.

### RNA extractions and microarray profiling.

Cultures of C. albicans strain SN152 were inoculated from a fresh colony and grown overnight in YPD at 30°C. Cultures were then diluted to an OD_595_ of 0.05 in 200 mL of fresh YPD. The culture was divided into four volumes of 50 mL and treated with the following agents for 12 h: 4 μg/mL FLC and 4 μg/mL FLC plus 1 μg/mL CZ66. Cells were then centrifuged for 2 min at 5,000 rpm, the supernatants were removed, and the samples were quick-frozen and stored at −80°C. RNA isolation was performed as described previously (59). RNA quality and integrity were checked using an Agilent 2100 bioanalyzer. Poly(A)-containing mRNA was isolated from the total RNA by poly(T) oligonucleotide-attached magnetic beads. The DNA probe was used to hybridize rRNA, RNase H selectively digested the DNA/RNA hybridization chain, and DNase I digested the DNA probe. After purification, the required RNA was obtained. The obtained RNA was fragmented and reverse transcribed by the random N6 primers, and then the cDNA double strand was synthesized to form double-stranded DNA. The end of the synthesized double-stranded DNA was flattened and phosphorylated at the 5′ end; the 3′ end formed a sticky end with a protruding “A” and then connected to a bubbly joint with a protruding “T” at the 3′ end. PCR amplified the ligation products with specific primers. The PCR product was thermally denatured into a single strand, and then a bridge primer was used to cyclize the single-strand DNA to obtain a single-strand circular DNA library and then sequenced (BGI Genomics, China).

After obtaining the raw data by sequencing, we trimmed reads with low quality (a base with a mass value of fewer than 15 accounts for more than 20% of the total base of the read), joint contamination, and high content of N (>5%) of an unknown base to obtain clean reads by the software SOAPnuke (BGI Genomics, China). Then, we mapped the reads by the software HISAT (Hierarchical Indexing for Spliced Alignment of Transcripts [http://www.ccb.jhu.edu/software/hisat]) (62). Bowtie2 was used to compare clean reads to the reference gene sequence (63), and then RSEM was used to calculate the expression level of genes and transcripts (64). The comparative analysis was carried out by a DEseq2 method (65). Assays were performed in duplicate three times.

### Quantitation of the percentage of ergosterol.

The effects of CZ66 and the Erg251 on ergosterol biosynthesis in C. albicans were determined by a method described previously with slight modifications (66). Briefly, 1 mL of YPD with or without the indicated drugs was added to 100 mL of different strains and incubated at 30°C for 12 h at 200 rpm. Cells were collected and washed three times with double-distilled water (ddH_2_O), and the weight of the wet cell pellet was adjusted to 0.7 g. Furthermore, 2.5 mL of ddH_2_O and 6 mL of 15% NaOH resolved in 90% ethanol were added to each pellet and mixed thoroughly. The suspension was incubated at 80°C for 1 h in a water bath. Sterols were extracted by adding 18 mL of petroleum ether in total three times, and each time the mixture was vortexed vigorously for 2 min and allowed to stand for 3 min. The petroleum ether layer was transferred to the same clear glass tubes and washed twice with ddH_2_O. The collected petroleum ether was volatilized at 65°C for 20 min in the water bath. Finally, the sterols were extracted by the addition of 600 μL of *n*-hexane, and a 200-μL aliquot was injected for gas chromatography-mass spectrometry (Finnigan Voyager, USA) with HP-50 columns (50% phenyl–50% methylpolysiloxane, 30 m by 0.25 mm by 0.25 μm). Before injection, 20 mg of cholesterol was added to each sample as the internal reference for quantification of other sterols. Sterols of interest were identified by their relative retention times, and mass spectra were compared with the sterol profiles of NIST. Assays were performed in duplicate three times.

### Rhodamine 6G efflux.

A previously described protocol was used to determine rhodamine 6G efflux (67). Briefly, 1 mL of overnight C. albicans culture was transferred to 100 mL of fresh YPD medium. A 1-μg/mL concentration of CZ66 was added, and the same volume of DMSO was added to the control group. After continuous culture at 30°C at 200 rpm and shaking for 6 h, the cells were pelleted and washed three times with phosphate-buffered saline (PBS) without glucose. The C. albicans cells were resuspended in PBS solution, adjusted to 1.0 × 10^8^ cells/mL, and grown for 1 h at 30°C to fully exhaust the glucose. Rhodamine 6G was added to each tube to make its final concentration of 10 μM, and cells were grown for 30 min at 30°C to saturate the absorption of rhodamine 6G. The cells were washed and resuspended in PBS with glucose (2 mM) or without glucose. An aliquot of 1 mL was taken after 0, 20, 40, and 60 min, respectively, and centrifuged at 10,000 rpm for 2 min. The absorbance of the supernatant was measured at 527 nm. Assays were performed in duplicate three times.

### Transmission electron microscopy analysis.

The membrane damage caused by the deletion of the *ERG251* gene and the *ERG3* gene or by FLC plus CZ66 were imaged by transmission electron microscopy. Briefly, 100 μL of C. albicans cultured in YPD medium overnight were added to 10 mL fresh YPD medium with or without indicated FLC or CZ66 and incubated at 30°C for 12 h with shaking at 200 rpm. After centrifugation, cell pellets were fixed with 2.5% glutaraldehyde at 4°C for 24 h. After that, the pellets were dropped onto the copper grid with carbon film for 3 to 5 min; 2% phosphotungstic acid was dropped on the copper grid to stain for 1 to 2 min; filter paper was used to absorb excess liquid and dry at room temperature. The copper grids were observed under a Hitachi H-7800 transmission electron microscope and images were obtained. The ultrastructures of strain SN152, the *ERG25*1 gene null mutant, and the *ERG3* gene null mutant were compared to assess the effects of compounds.

### Molecular docking and molecular dynamics simulations.

For details of Erg251 and CZ66 interaction by molecular docking and molecular dynamics simulation, see Text S1.

### Cytotoxicity tests.

The cytotoxic effect of CZ66 on the HUVECs’ viability was assessed by the CCK-8 assay (68). Briefly, HUVECs (1 × 10^4^ cells/well) were seeded in 96-well plates and cultured in a Dulbecco’s modified Eagle’s medium supplemented with 10% fetal bovine serum (FBS) at 37°C for 3 h for adhesion. After incubation, the cell supernatant was removed, and different concentrations of CZ66 (from 64 to 0.125 μg/mL) dissolved in fresh medium without FBS were added. The plates were incubated at 37°C for 24 h. The CCK-8 solution was added to each well (10 μL/well) and incubated at 37°C for 2 h. The absorbance at 450 nm was measured using an enzyme-linked immunosorbent assay (ELISA) plate reader. Cells incubated in a DMEM with 0.1% DMSO were set as the standard for 100% viability. For each concentration of CZ66, the mean values of the mean absorbance rates from three wells were calculated.

### Western blot analysis.

Cells were incubated at 30°C for 16 h in the presence (1 μg/mL) or absence of FLC. Cells were washed with sterile water once and then were subjected to cell lysis using the Bead Ruptor12 system (OMNI International, USA) in lysis buffer (PBS [pH 7.2 to 7.6]) containing 5 mM EDTA (pH 8.0), 1 mM phenylmethylsulfonyl fluoride (PMSF), and 1.0% protease inhibitor cocktail (Sigma). Proteins were separated by 4 to 20% SDS-PAGE and transferred to a polyvinylidene difluoride (PVDF) membrane. After blocking, anti-GFP antibody (Santa Cruz) or antitubulin antibody (Abbkine) was used for probing GFP-tagged proteins or tubulin, which were then detected using the secondary antibody goat anti-mouse IgG conjugated with horseradish peroxidase (HRP) (Santa Cruz) and the Pierce ECL system (Thermo Fisher Scientific, MA, USA).

### Systemic murine candidiasis model.

Groups of BALB/c female mice (6 to 8 weeks) were inoculated via lateral tail vein with 200 μL PBS containing 2 × 10^5^ C. albicans cells. FLC and CZ66 were administered to the infected mice at a dose of 1 mg/kg once a day intraperitoneally for 3 days starting 2 h after the injection with C. albicans. Mice were monitored daily for survival for a period of 31 days. Kaplan-Meier analyses were used to indicate the survival probabilities, and log rank testing was used to evaluate the significance of survival curves.

All experimental procedures involving animals were approved by the Shanghai Tenth People's Hospital Animal Care Committee (no. SHDSYY-2020-G005).

### Data availability.

The RNA sequencing data reported in this study were archived in the Sequence Read Archive (SRA) under accession no. PRJNA880988.
