# Supplementary figures and images for: A Small Molecule Inhibitor of Erg251 Makes Fluconazole Fungicidal by Inhibiting the Synthesis of the 14α-Methylsterols
Source: mBio. 2022 Dec 8;14(1):e02639-22. doi: 10.1128/mbio.02639-22 (PMC9973333; doi:10.1128/mbio.02639-22)

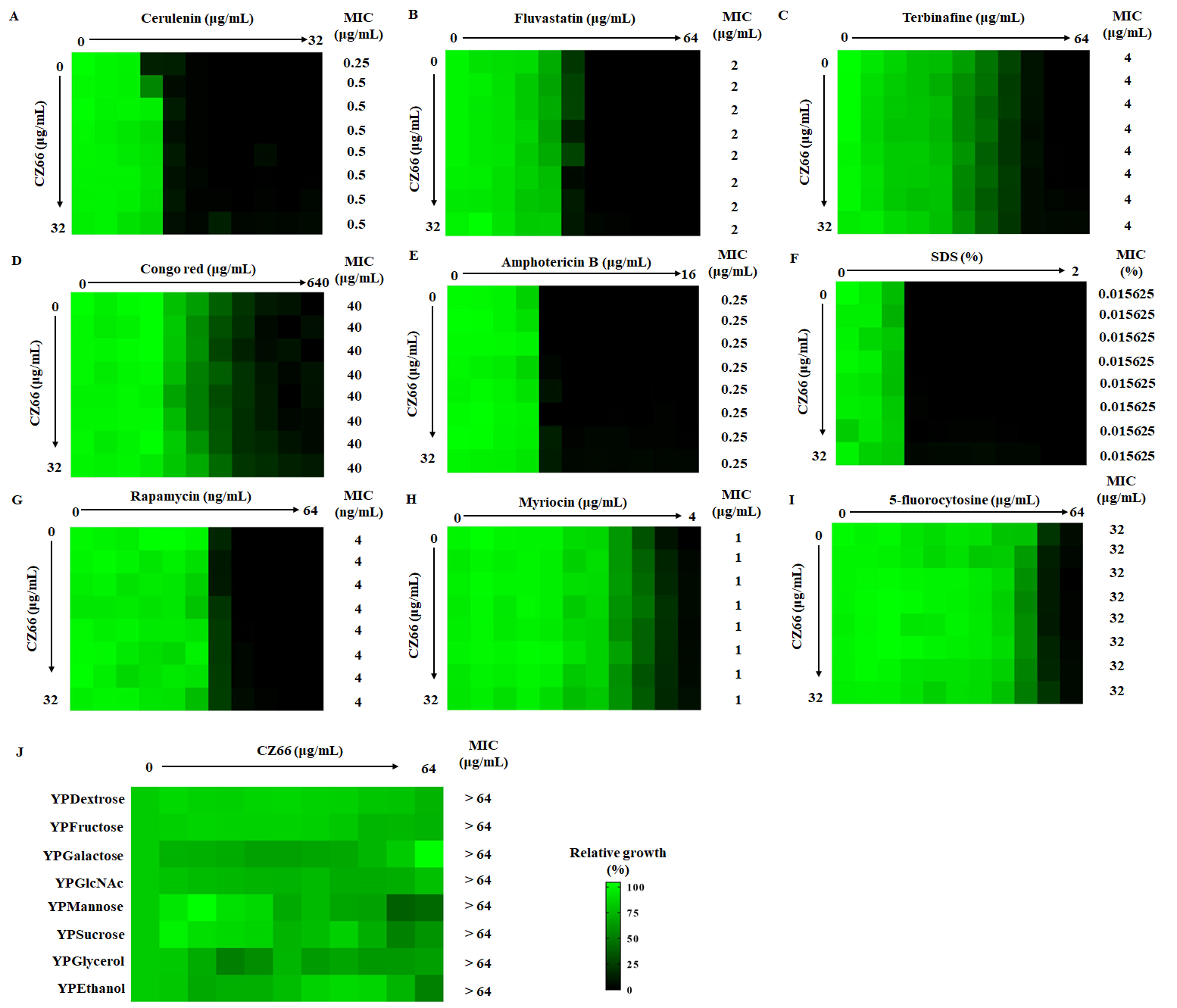

Supplement: FIG S1 [file mbio.02639-22-s0001.tif]

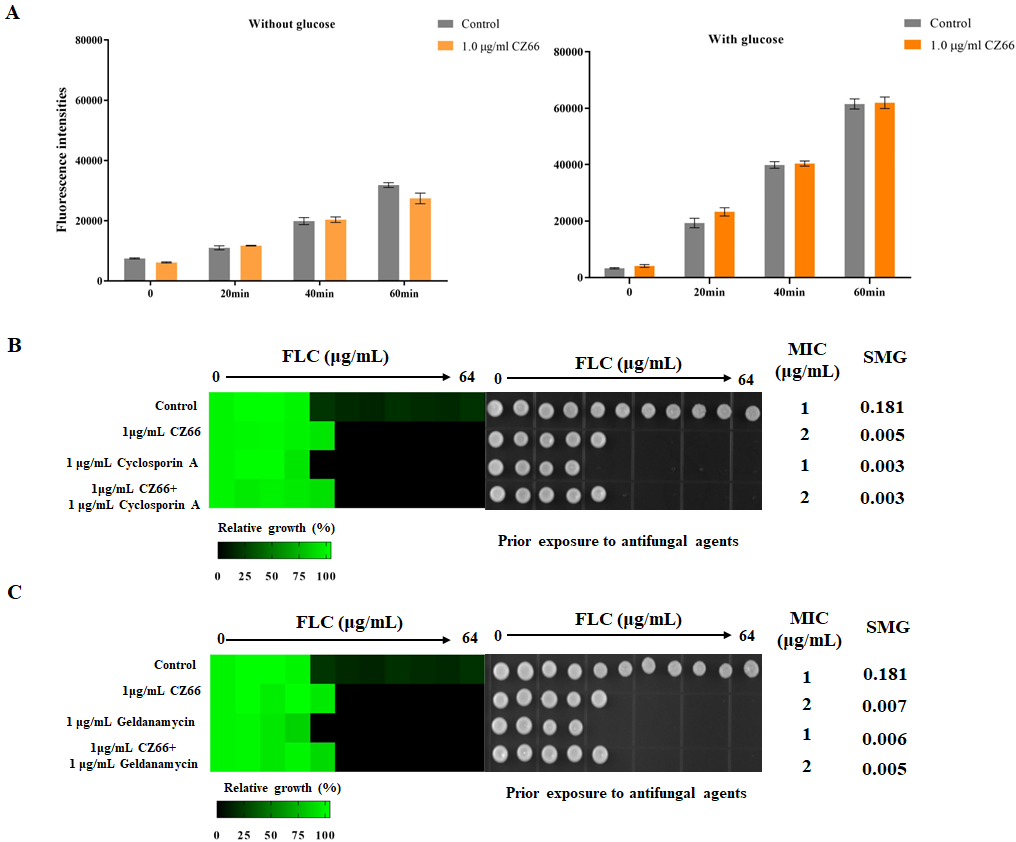

Supplement: FIG S2 [file mbio.02639-22-s0002.tif]

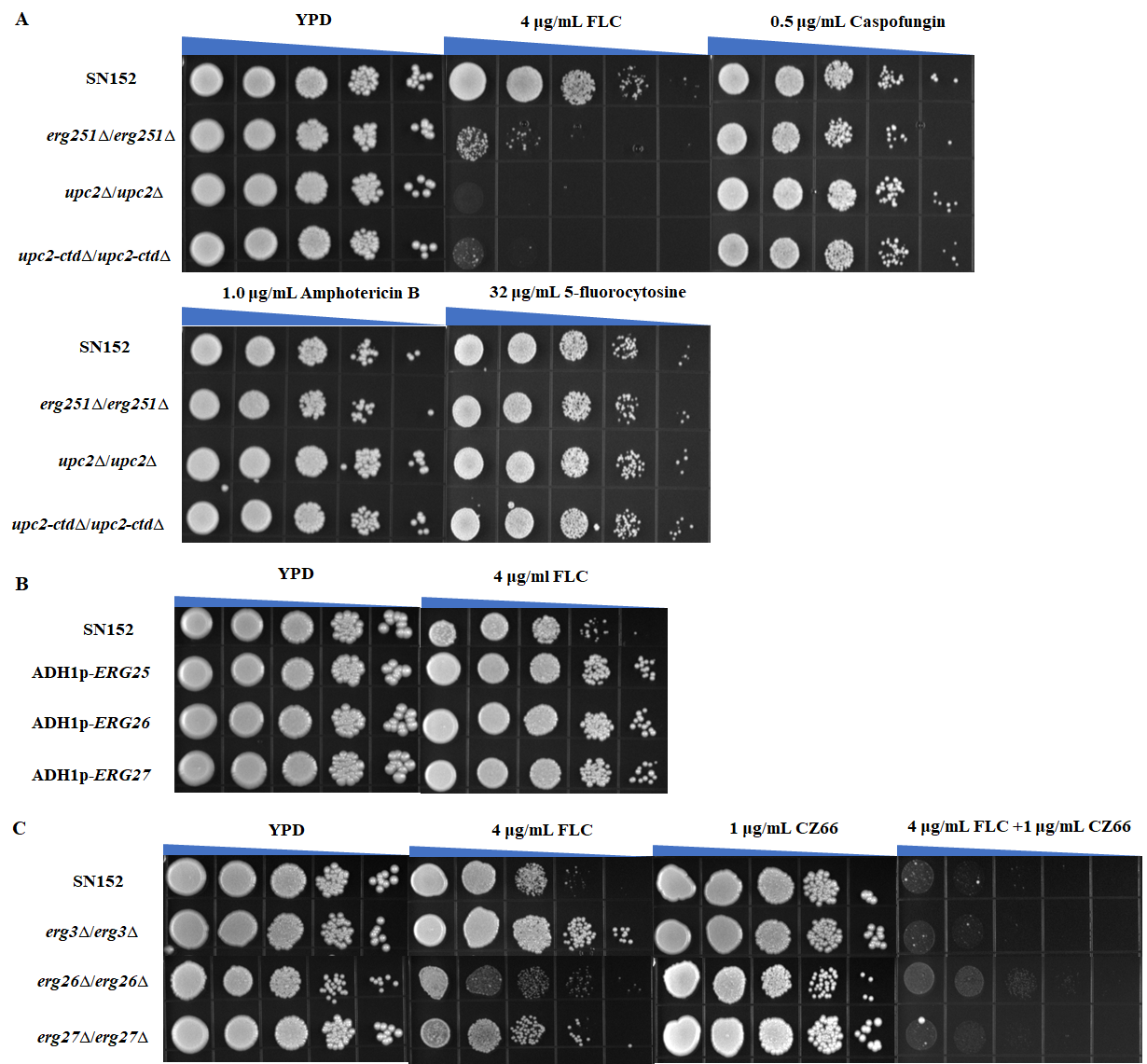

Supplement: FIG S3 [file mbio.02639-22-s0003.tif]

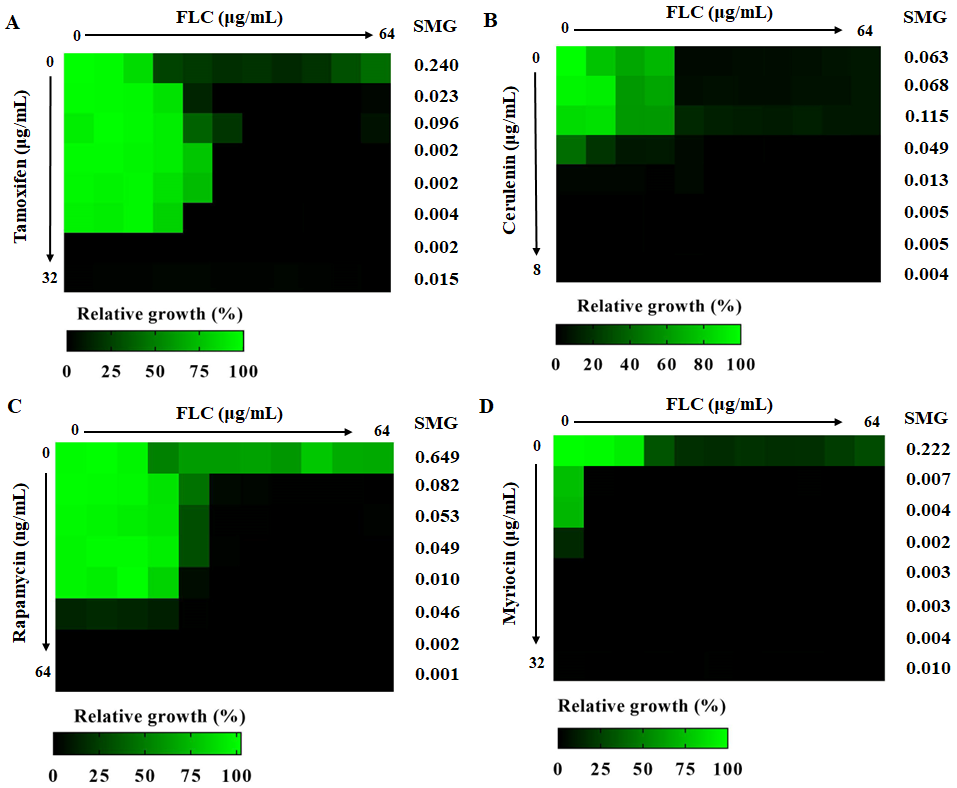

Supplement: FIG S4 [file mbio.02639-22-s0004.tif]

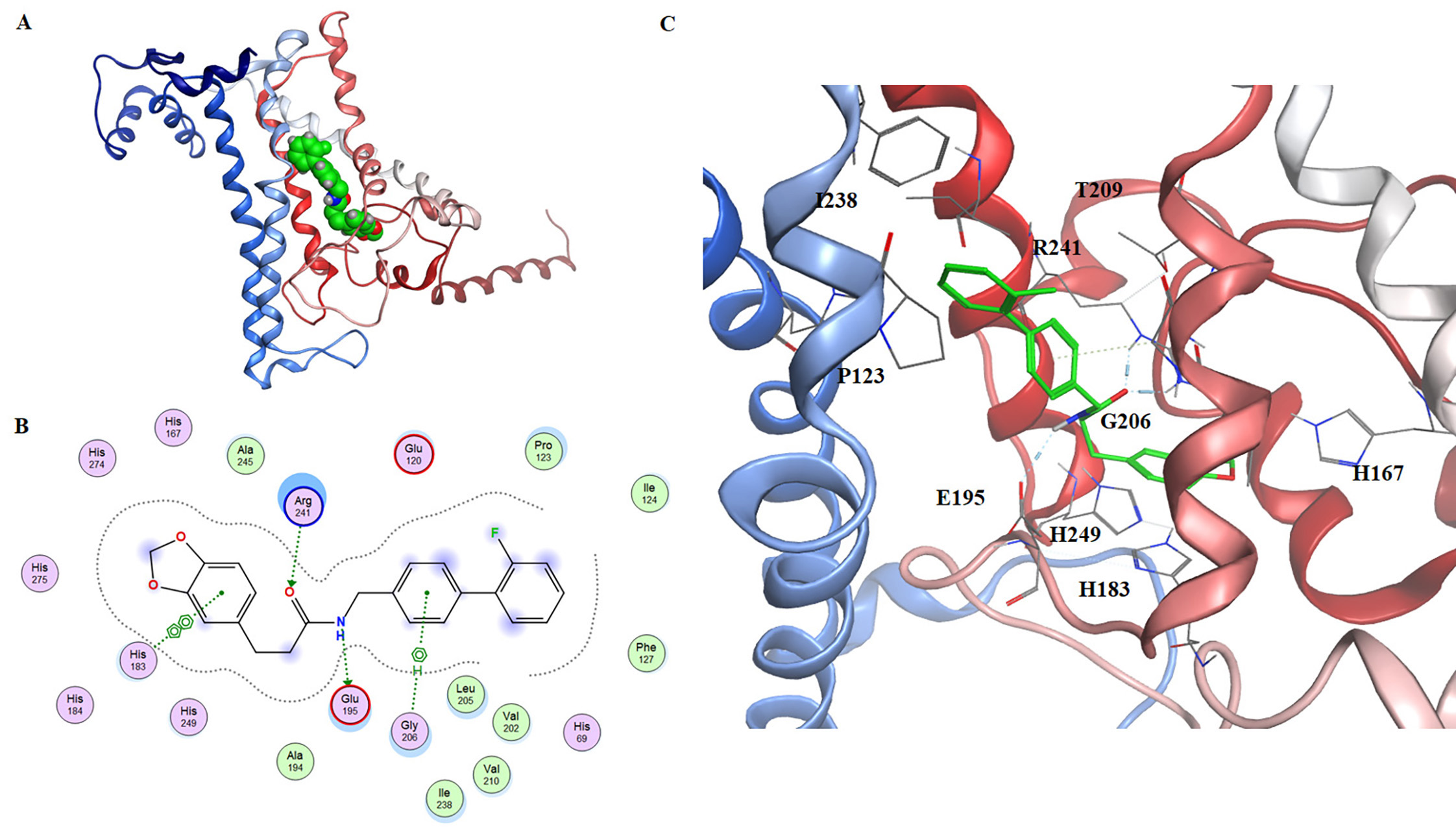

Supplement: FIG S5 [file mbio.02639-22-s0005.jpg]

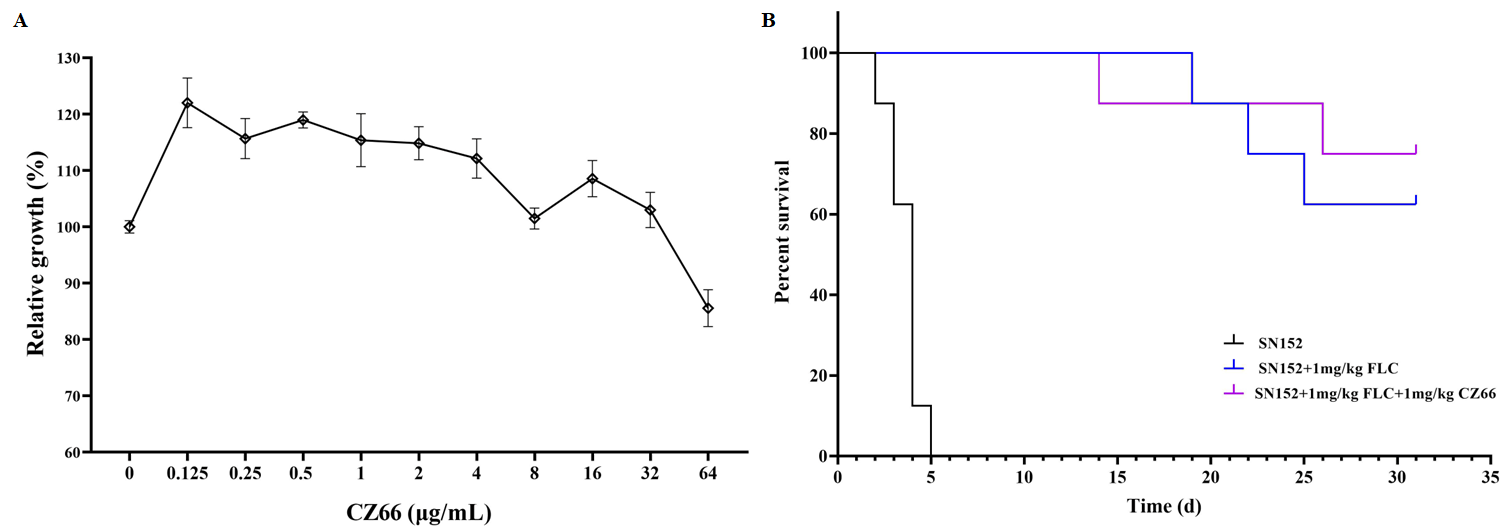

Supplement: FIG S6 [file mbio.02639-22-s0006.tif]
