# Supplementary material for: A Small Molecule Inhibitor of Erg251 Makes Fluconazole Fungicidal by Inhibiting the Synthesis of the 14α-Methylsterols
Source: mBio. 2022 Dec 8;14(1):e02639-22. doi: 10.1128/mbio.02639-22 (PMC9973333; doi:10.1128/mbio.02639-22)
Supplement: TEXT S1 [file mbio.02639-22-s0010.docx]

Erg251-CZ66 interaction analysis by molecular dynamics simulation

**Introduction of *Candida albicans* Erg251 and CZ66**

Although the sequence of *C. albicans* Erg251 was identified, more detailed information about the protein has currently not been unrevealed. Sequential BLAST analysis showed that *C. albicans* Erg251 shared a 99.7% sequence similarity with yeast C-4 methylsterol oxidase, which indicates the protein may have potential with C-4 methylsterol oxidase activity. *C. albicans* Erg251 contains 321 amino acids (M1-L321), and the fatty acid hydroxylase domain is defined as the region of A153-T295. The 3D structure of the protein has not been resolved so far. Therefore, AlphaFold (<https://alphafold.ebi.ac.uk/entry/A0A1D8PLB5>) was used to predict the 3D structure (Figure 1) (1). The 3D model of Erg251 was well packed and five helix bundled together to maintain the potential fatty acid hydroxylase or oxidase activity.


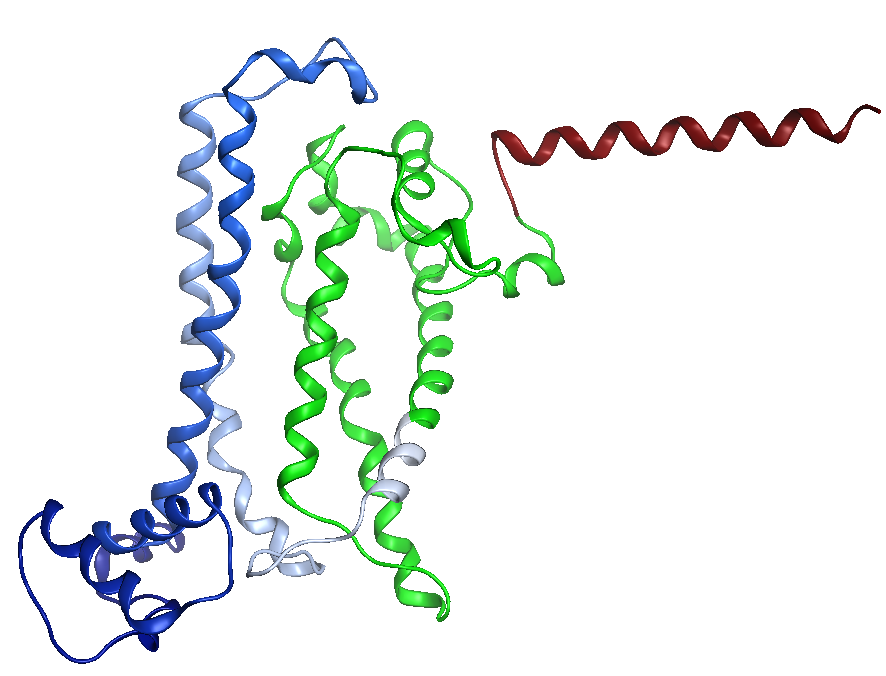


**Figure 1.** 3D structure of *C. albicans* Erg251 based on AlphaFold. N to C terminus (blue to red). The fatty acid hydroxylase domain was indicated by green.

The compound CZ66 ([CHEMBL4085632](https://www.ebi.ac.uk/chembl/compound_report_card/CHEMBL4085632)) has chemical properties such as a molecular weight of 377 Dal, polar surface area of 47.5 Å^2^, rotatable bonds of 6, and hydrogen bond donor of 1, was selected as a ligand in the molecular docking process (Figure 2).


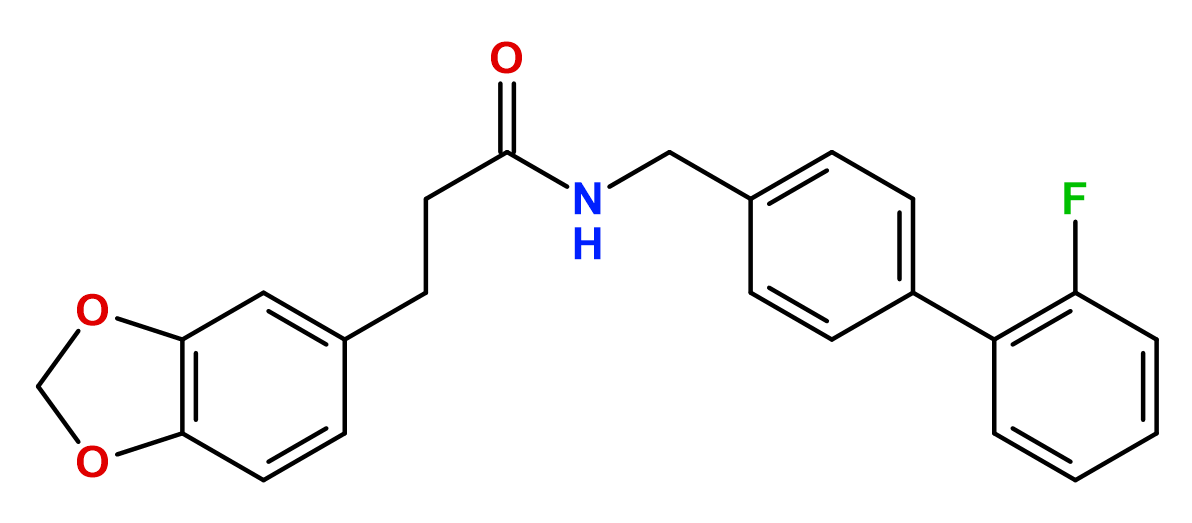


**Figure 2.** The chemical structure of CZ66 (CAS:4773-96-0).

**Molecular docking**

**Selection of the 3D structure of Erg251 and determination of the docking area**

As described previously, the 3D structure of Erg251 was built by Alphafold, and the 3D structure was then processed by protonation and optimization by MOE software (version 2020.9). Two small molecular binding pockets were detected using the 'SiteFinder' plugin in MOE software (version 2020.9), and the properties of the two pockets are shown in Figure 3 (2). Pocket1 contained 51 hydrophobic atoms and maximally accommodated 139 atoms, with a total propensity for ligand binding score (PLB) of 2.83. Pocket1 was formed by the residues such as Phe65, His69, Val119, Glu120, Pro123, Ile124, Leu126, Phe127, Val130, Glu159, His163, His167, His180, His183, Ala194, Glu195, Ala197, Glu201, Val202, Met203, Leu205, Gly206, Val207, Thr209, Val210, Ile238, Arg241, Leu242, Gln244, Ala245, Ser248, His249, His271, and Ser284. Pocket2 contained 30 hydrophobic atoms and maximally accommodated 83 atoms, with a total propensity for ligand binding score (PLB) of 2.75. Pocket2 was formed by the residues such as Arg76, Trp80, Phe156, Leu240, Phe243, Gln244, Asp247, Ser248, Ser250, Tyr252, Asp253, Phe254, Trp256, Ser257, Leu258, Asn259, Trp265, Ala266, Gly267, Ala268, Ala269, Ser284, and Ser285.


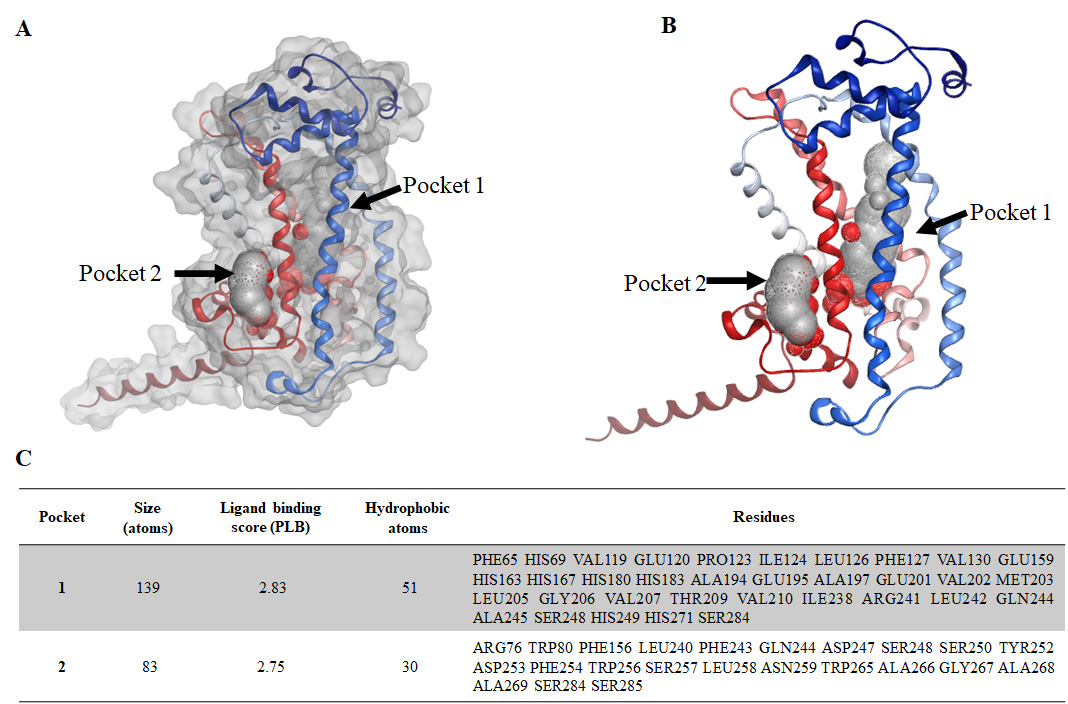


**Figure 3.** Identification of the ligand-binding pockets for Erg251. **(A)** The pockets were shown in the molecular surface of Erg251 and **(B)** in the new cartoon version. **(C)** The property of the pockets.

**The 3D structure construction and multiple conformer generation**

The plugin' builder tool' of MOE software (version 2020.9) was used to create the molecular structure of CZ66, and the 3D structure of CZ66 was generated by using energy minimization (Figure 2). The multiple conformations of the compound were generated by using the 'conformation search' program of MOE software (3). The parameters used for conformation generation were shown (Figure 4). Finally, 96 conformations were generated for CZ66.


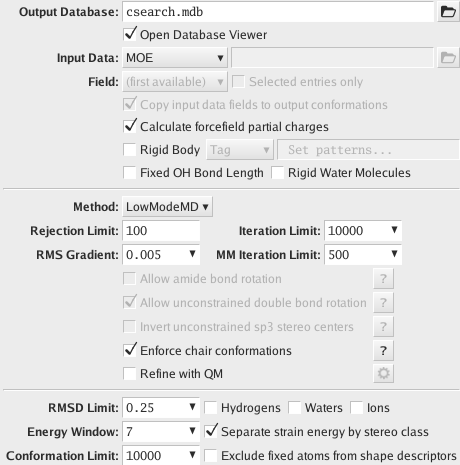


**Figure 4**. The parameters for multiple conformation generation.

**Molecular docking and Docking results.**

The 3D structure of Erg251 was initially constructed using AlphaFold, and the structure was then protonated under the AMBER10:EHT force field. Both pocket1 and pocket2 were chosen as docking areas (Figure 3). InducedFit docking protocol was used for the flexible docking process, with the parameters as the Triangle Match algorithm for docking mode generation and the London δG scoring function for the binding energy calculation (4). The docked poses were then optimized under the Induced Fit algorithm, and the GBVI/WAS δG scoring function was eventually used to calculate the final binding affinity of the optimized docking poses. Finally, the best ten docked modes of CZ66 for each docking area were retained (Figure 5).


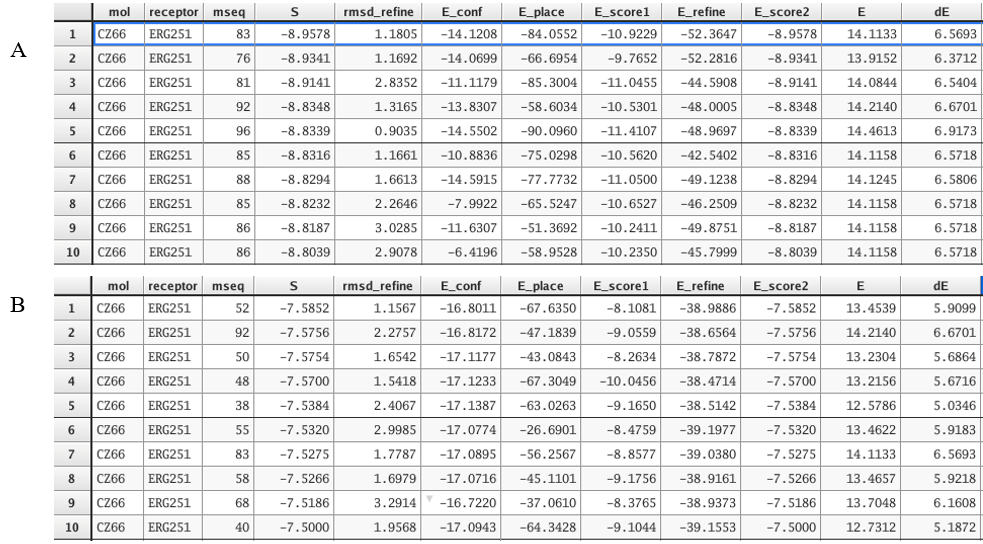


**Figure 5.** The docking scores of 10 poses for CZ66 in Pocket1 (A) and Pocket2 (B). E_Conf is the energy of the conformer, E_Place is the energy generated during the placement, E_Score1/2, E_refine, etc., are the values of the van der Waals force, electrostatic effect, and solvent effect. The S value indicated the interaction energy between protein and compound.

As shown in Figure 5, the docking scores of the ten poses of CZ66 for the two pockets were calculated, where E_Conf was the energy of the conformer, E_Place was the energy generated during the placement, E_Score1/2, E_refine, etc. were the values of the van der Waals force, electrostatic effect, and solvent effect. CZ66 had an averaged lower binding energy in the pocket1 area than that in the pocket2 area, which indicates that CZ66 was preferable to bind Erg251 in the pocket1 area. Therefore, in the following MD simulation study, the docked pose of CZ66 in the pocket1 area was selected as starting point.

The S value indicated the binding energy between Erg251 and CZ66, where the smaller the value, the stronger the interaction. Based on the S value, the best docking pose of CZ66 was selected for the follow-up molecular dynamics simulation (Figure 6).


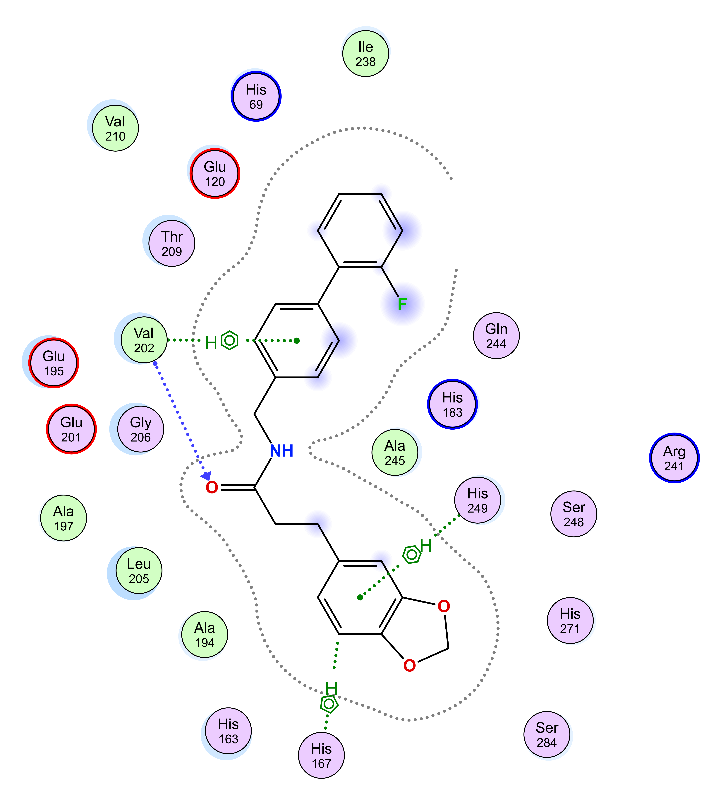


**Figure 6.** Schematic diagram of the interaction between protein Erg251 and CZ66.

The binding energy was -8.95 kcal/mol in the selected docked pose. The surrounding residues such as Val202, His249, His167, Glu201, Arg241, Leu205, Ala245, His69, Glu120, His163, Glu195, Gly206, and Gln244 contributed to the CZ66 interaction, where residue His167 and His249 formed two pairs of aromatic hydrogen bonds with CZ66, and Val202 formed a hydrogen bond with CZ66 (Figure 6).

**Molecular dynamics simulation**

**Erg251-CZ66 complex configuration for the molecular dynamics simulation**

The docked pose of the Erg251-CZ66 complex with the best docking score was selected as the initial structure for molecular dynamics simulation. GROMOS96 43a1 force field was used to construct the topology and coordinates for Erg251. The topological and 3D structures of CZ66 were constructed by PRODRG (http://davapc1.bioch.dundee.ac.uk/cgi-bin/prodrg/run.html.). The Erg251-CZ66 complex was dissolved with 44903 SPCE water molecules, and the final system was in a cubic box with a size of 1405.92 (nm^3^). According to the box's volume and the electronegativity of the protein, 130 Na^+,^ and 132 CL^-^ were added as counter ions to make the entire system electrically neutral.

**Parameter configuration of molecular dynamics simulation**

In this study, ten nanoseconds (ns) molecular dynamics simulation (MD) was performed by using GROMACS (version5.6) software package. The Erg251-CZ66 complex was first optimized by energy minimization (EM) with the steepest descent algorithm. The EM was finished when the maximum force of the system was less than 1000 newtons at 856 steps, and the potential energy of the system was -2.5×10^6^ kcal/mol. The system's temperature was increased to 300 K and controlled by the NVT protocols with a 100 picosecond (ps) simulation.

The Parrinello-Rahman algorithm was then used to control the system's pressure at 1 atm, and the temperature was at 300 K. During the ten ns MD simulation, the cutoff of short-range non-bonding interaction was set to 10 Å, and the long-range electrostatic interaction was treated with PME method. The SHAKE algorithm constrained all chemical bonds including hydrogen atoms, and the periodic boundary was selected for the MD simulation. The step size of the simulation was set to 2 femtoseconds (fs), and the coordinates were saved every two ps during the simulation.

The root mean square deviation (RMSD) and root mean square fluctuation (RMSF) of each residue in the system were analyzed with the initial structure as the reference. Every four frames from the MD trajectory were retrieved for the binding free energy calculation by using the MM-PBSA method. Every 50 frames from the MD trajectory were retrieved to analyze the interaction between Erg251 and CZ66 by using the PLIF plugin of MOE software (version 2020.09) with default parameters.

**The conformation deviation of the Erg251 during the MD simulation**

The RMSD plot showed that the 3D conformation of Erg251 was changing during the simulation, while CZ66 was relatively stable during the simulation (Figure 7). The structure of Erg251 was reaching stable at six nanoseconds, where the RMSD value was 0.48 and 0.12 nm for Erg251 and CZ66, respectively (Figure 7). Therefore, the following studies were based on the trajectories from six nanoseconds to 10 nanoseconds during the simulation.


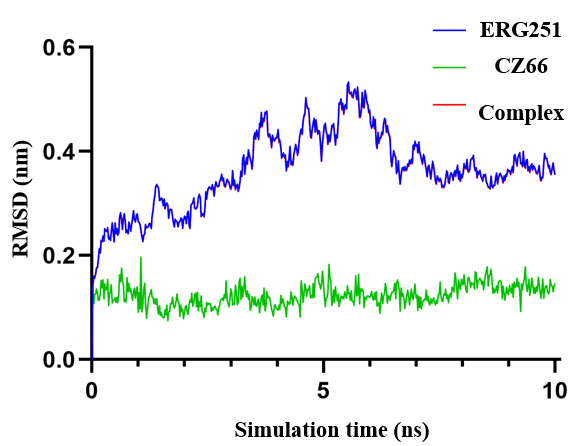


**Figure 7.** The RMSD plots of the system during the ten ns MD simulation. ERG251 was indicated by blue, CZ66 was indicated by green, and the complex was indicated by red.

The flexibility of each residue was analyzed by calculating the root mean standard fluctuation (RMSF) (Figure 8). Based on RMSF values, ten residues such as Gly62, Tyr73, Ala60, Ile136, Thr61, Cys77, His230, Leu242, Gly191, and Phe74 were identified as the most stable residues, which may contribute to the ligand interaction or the structure stabilization. In order to study the CZ66 surrounding residues, the minimum distance of each residue to CZ66 was calculated during the simulation. The results indicated that the surrounding residues were mainly located in five regions, as indicated by orange rectangles in Figure 8, and those residues were also structurally stable (with low RMSF values). Among these residues with the RMSF value less than 0.1 nm, nine residues such as Tyr73, Ile124, and Trp237 may be involved in the ligand-binding since their distances to CZ66 were less than 0.4 nm (Figure 8).


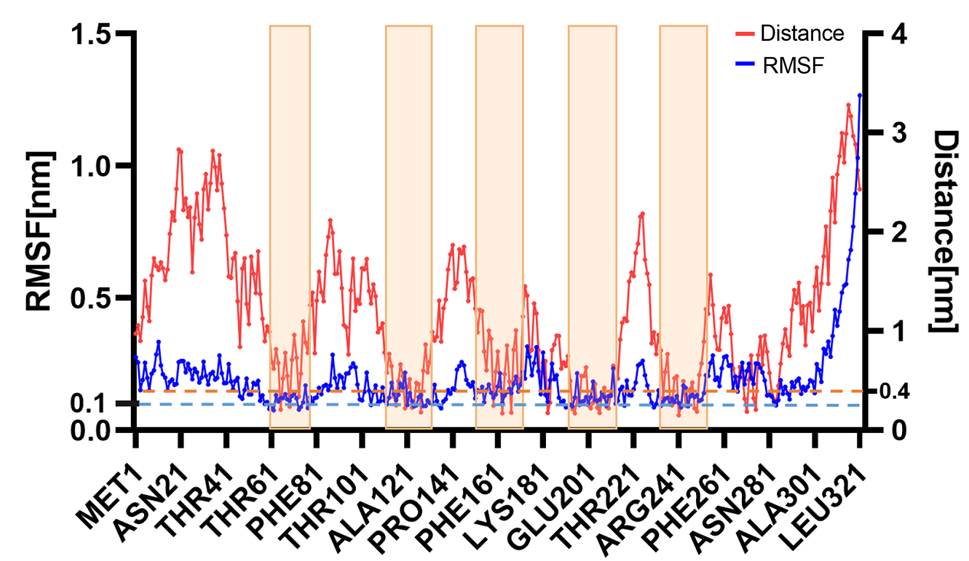


**Figure 8**. The Distance and RMSF curves during the MD simulation. The orange rectangle indicated the residues which may interact with the CZ66.

**The binding free energy between Erg251 and CZ66**

The binding free energy ΔG_bind_ of the complex was calculated as -236.239 kcal/mol by using MM-PBSA (Table 1) (5). The binding energy was divided into electrostatic interaction, van der Waals interaction, and non-polar solvation interaction, where the van der Waals interaction was the main contributor to the complex formation (Table 1). The rest contributors were electrostatic and non-polar solvation interactions with the value of -156.710 kcal/mol and -21.166 kcal/mol, respectively. However, the polar solvation free energy GPB was calculated as 175.278 kcal/mol, which indicated that polar solvation might impair the complex formation.

**Table 1.** **Binding free energy of Erg251-CZ66 complex system.**

| **Energy**  **(kcal/mol)** | **E_MM_** | | **G_solv_** | |  |
| --- | --- | --- | --- | --- | --- |
|  | **E_ele_** | **E_vdw_** | **G_PB_** | **G_nonp_** | **ΔG_bind_** |
|  | -156.710 | -233.640 | 175.278 | -21.166 | -236.239 |

**The binding free energy decomposition**

To explore the main contributors to the interactions between Erg251 and CZ66, we used energy decomposition to identify the critical residues for Erg251-CZ66 interaction. Six residues of Erg251 were identified as the main contributors with binding energy stronger than -3 kcal/mol, which were Val202, Glu159, Glu201, Ile238, Leu205, and Arg241 (Figure 9).


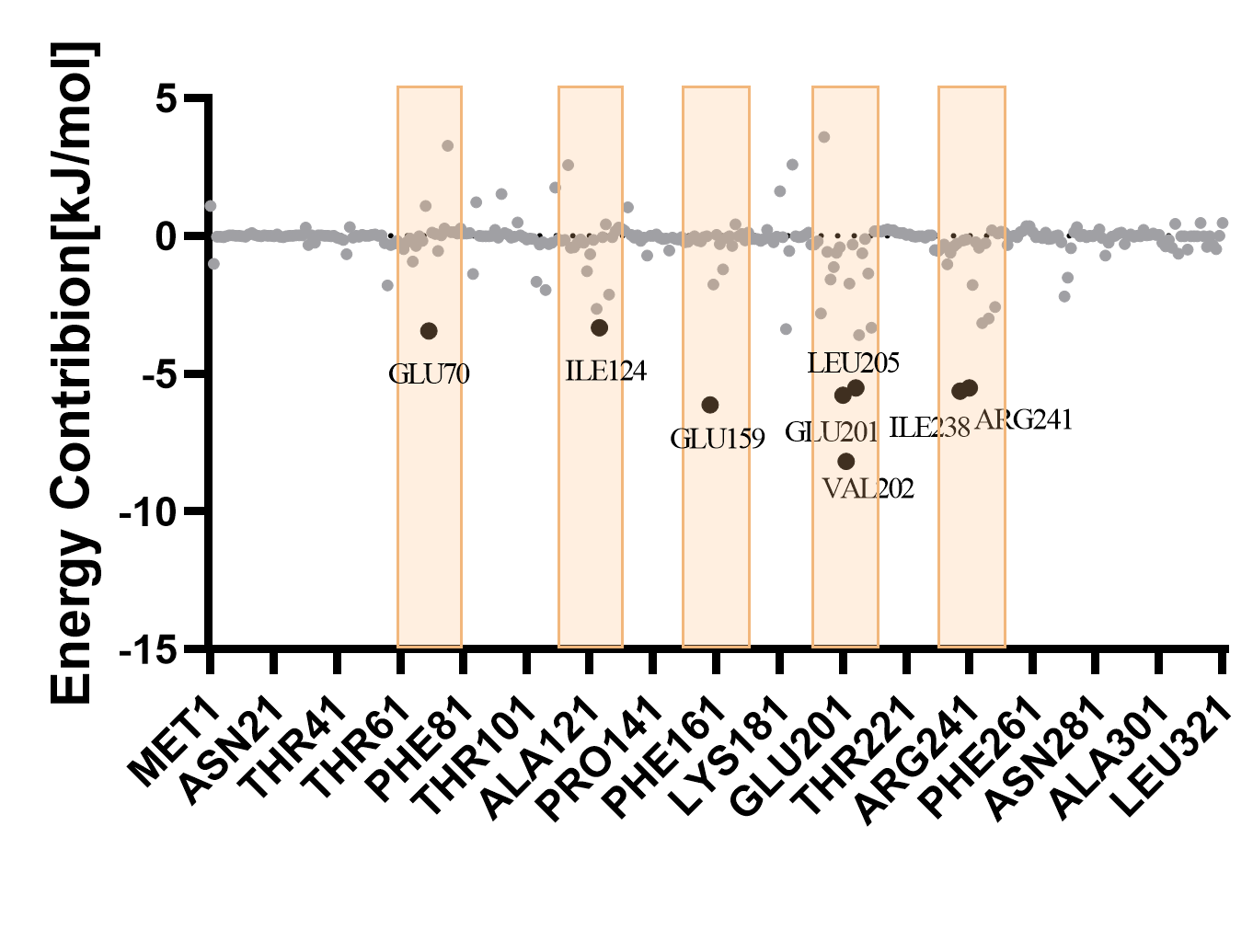


**Figure 9**. The energy contribution of each residue in Erg251. The key residues for binding energy were labeled, and the orange rectangle indicated the residues which may interact with the CZ66.

**The protein-ligand interaction interface fingerprint (PLIF) between Erg251 and CZ66**

The complex structures were extracted every 50 frames. And the interactions between CZ66 and Erg251 were analyzed by the PLIF module of MOE (2020.09). PLIF showed that the contact residues changed during the MD simulation (Figure 10), where residues such as Arg241and Glu195 interacted with CZ66 at a high frequency during the simulation.


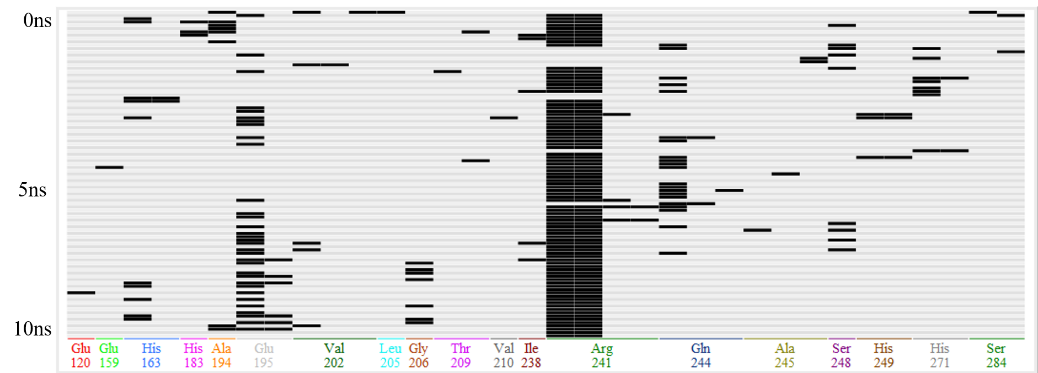


Figure 10 Protein-ligand interaction interface fingerprints between CZ66 and Erg251 during the simulation.

During the ten ns MD simulation, CZ66 frequently interact with 19 residues such as His163, Glu195, and Arg241. However, in the unstable stage (0-6 ns), the residues such as Gln244, Ser248, and His271 formed interactions with CZ66, while after six ns simulation, CZ66 frequently interacted with GLY206 (Figure 10). Based on the contact frequency, we concluded that CZ66 was most likely to interact Erg251 with Glu195, Arg241, and Glu206, such as the complex at the simulation of 10 ns (Figure 11). His183 and Gly206 formed aromatic hydrogen bonds in the complex, and Arg241 and Glu195 formed hydrogen bonds with CZ66 (Figure 11).


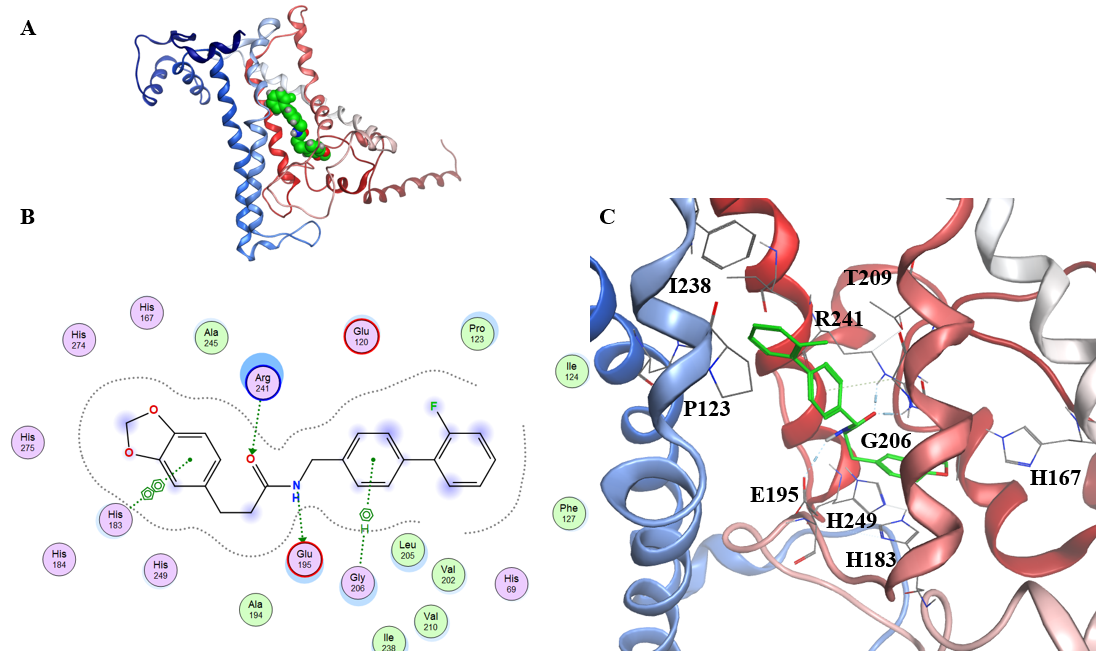


**Figure 11.** The representative binding pose between Erg251 and CZ66 during the simulation. **(A)** CZ66 (green) was located in the binding area of Erg251. **(B)** The 2D interaction diagram between CZ66 and the residues of Erg251. **(C)** CZ66 interacted with the residues in the interface of Erg251.

**Conclusion**

This study attempted to study the interaction between Erg251 and CZ66 using molecular docking and MD simulation. The 3D structure of *C. albicans* Erg251 was constructed by AlphaFold because the protein's crystal structure was not resolved. Two pockets were used as docking areas, and the multiple conformations of CZ66 were used as the docking ligands. The flexible docking protocol was used to perform the docking process. After the molecular docking process, the complex with the best docking score was selected as the initial structure for a ten ns MD simulation. When the system reached stability, several properties such as the RMSF value, minimum distance, binding energy, and energy decomposition were calculated, and more information about the interaction was retrieved. We found Gly62, Tyr73, Ala60, Ile136, Thr61, Cys77, His230, Leu242, Gly191, and Phe74 to be the most stable residues. Tyr73, Ile124, and Trp237 were the closest residues to CZ66. The van der Waals interaction was the main contributor to the complex formation, but polar solvation free energy may impair the complex formation. Six residues, Val202, Glu159, Glu201, Ile238, Leu205, and Arg241, favored the ligand binding. Although 19 residues were propensity to the ligand interaction, three Glu195, Gly206, and Arg241 were the common contact residues with the high frequency.

1. **References**

1. David A, Islam S, Tankhilevich E, Sternberg MJE. 2022. The AlphaFold Database of Protein Structures: A Biologist's Guide. J Mol Biol 434:167336.

2. Volkamer A, Griewel A, Grombacher T, Rarey M. 2010. Analyzing the topology of active sites: on the prediction of pockets and subpockets. J Chem Inf Model 50:2041-52.

3. Labute P. 2010. LowModeMD--implicit low-mode velocity filtering applied to conformational search of macrocycles and protein loops. J Chem Inf Model 50:792-800.

4. Naim M, Bhat S, Rankin KN, Dennis S, Chowdhury SF, Siddiqi I, Drabik P, Sulea T, Bayly CI, Jakalian A, Purisima EO. 2007. Solvated interaction energy (SIE) for scoring protein-ligand binding affinities. 1. Exploring the parameter space. J Chem Inf Model 47:122-33.

5. Kumari R, Kumar R, Open Source Drug Discovery C, Lynn A. 2014. g_mmpbsa--a GROMACS tool for high-throughput MM-PBSA calculations. J Chem Inf Model 54:1951-62.
